# Microtubule-associated protein 1S phosphorylation regulates dynein-driven transport and synaptogenesis in neurons

**DOI:** 10.1172/JCI188241

**Published:** 2026-09-15

**Authors:** André T. Lopes, Ondine Janiv, Suzanne Claxton, Sila K. Ultanir

**Affiliations:** Kinases and Brain Development Lab, The Francis Crick Institute, London, United Kingdom.

**Keywords:** Development, Neuroscience, Cytoskeleton, Neurodevelopment, Synapses

## Abstract

Cyclin-dependent kinase like 5 (CDKL5) is a serine-threonine kinase enriched in the mammalian brain whose loss of function causes a severe developmental and epileptic encephalopathy named CDKL5 Deficiency Disorder. We previously showed that CDKL5 phosphorylates the microtubule-associated protein MAP1S, but how this regulates microtubule-dependent functions is not well understood. To address this question, we generated MAP1S phosphomutant mice in which the CDKL5 phosphorylation sites S786 and S812 were mutated to alanine (MAP1S S786/812A; MAP1S SA). Using a microtubule cosedimentation assay, we found that dynein binding to microtubules was reduced in MAP1S SA and CDKL5 knockout (KO) brain lysates, and time-lapse imaging showed impaired dynein motility in dendrites from both genotypes. MAP1S SA mice also exhibited reduced AMPA receptor transport, dendritic spine density, and excitatory synapses, accompanied by anxiety-like behavior and motor, social, and memory deficits relevant to CDD. Mechanistically, MAP1S SA and CDKL5 KO neurons showed increased microtubule stability and reduced tubulin tyrosination, consistent with excessive MAP1S-mediated stabilization. Restoring tubulin tyrosination by expressing tubulin-tyrosine ligase rescued dynein transport defects. Together, these findings identify MAP1S phosphorylation as a critical regulator of microtubule dynamics and dynein-dependent transport.

## Abstract 2

This research work reveals for the first time that tubulin tyrosination and dynein-mediated transport deficits to be prominent in the absence of CDKL5 function.

## Graphical abstract

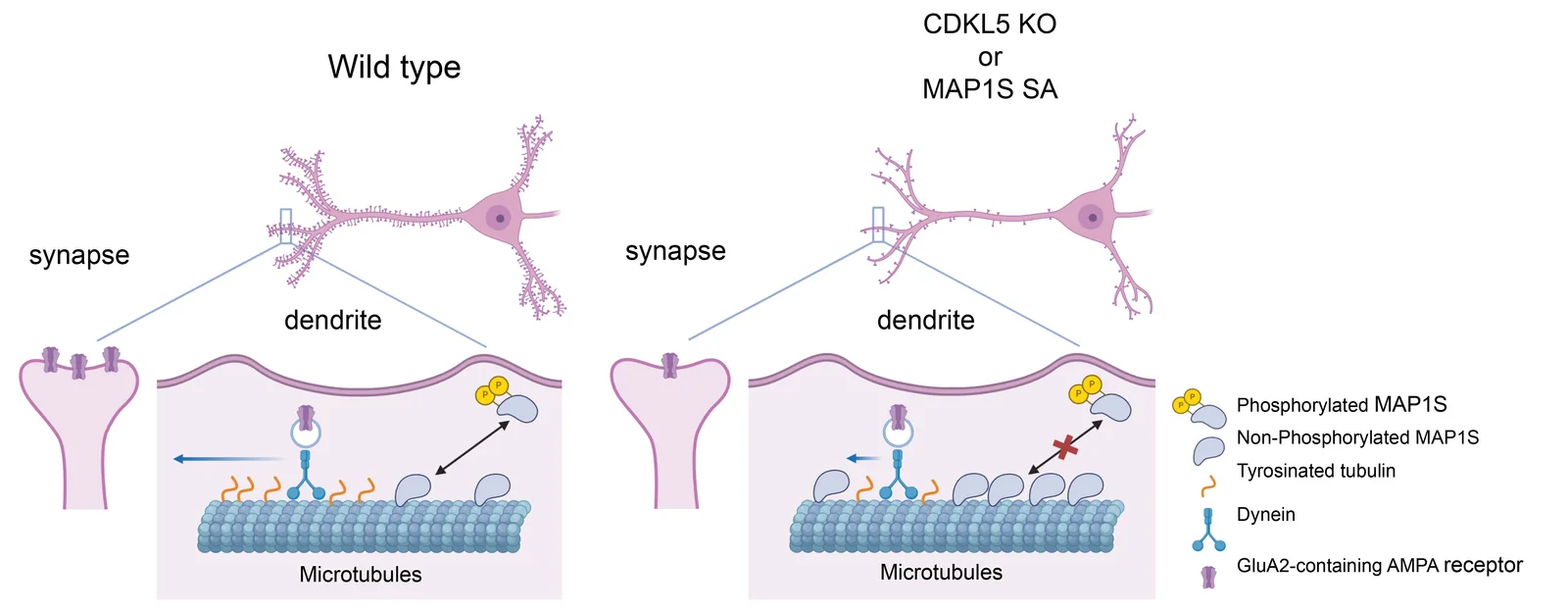

## Introduction

Cyclin dependent kinase-like 5 (CDKL5) is a 115 kDa serine/threonine kinase highly enriched in the brain, with expression starting at late embryonic development in rodents and humans (1–6). CDKL5 is on the X chromosome, loss-of-function mutations of CDKL5 cause CDKL5 deficiency disorder (CDD, OMIM 300203, 300672) (7–12). CDD is a developmental and epileptic encephalopathy characterized by intractable early-onset seizures, severe neurodevelopmental delay, intellectual disability, and autistic features (7, 13–16). CDD is more common in females than males (at a ratio of 4:1) and affects 1 in 40,000–60,000 live births each year, being one of the most common types of epilepsy in childhood (17–20). The vast majority of pathogenic missense mutations of CDKL5 are found in the catalytic domain (21, 22) and they reduce the kinase activity (23), highlighting its importance. There are currently no effective treatment options for this disorder, with patients with CDD being medicated for seizures and associated comorbidities.

CDKL5 is a kinase with an important role in the formation and maturation of neurons, cilia elongation, dendritic spine development, and synaptic plasticity (24–33). Using chemical genetics method, 3 microtubule-binding proteins, EB2, ARHGEF2, and MAP1S were identified as substrates of CDKL5, underscoring the importance of this kinase as a potential regulator of microtubule function (4). Despite several studies converging on the functional link between CDKL5 and neuronal cytoskeleton (4, 23, 34, 35), how CDKL5 regulates microtubule function at the molecular level remains poorly understood.

MAP1S is a direct CDKL5 substrate that it is widely expressed in the brain. MAP1S is a shorter homologue of MAP1A and MAP1B, and full-length MAP1S is posttranslationally cleaved into heavy chains and light chains (36–38). CDKL5 phosphorylates MAP1S at the N-terminal of its light chain. Upon phosphorylation of serines Ser786 and Ser812, MAP1S will detach from the microtubules and become more soluble in the cytosol (4). Despite this biochemical evidence, the role of MAP1S phosphorylation in regulating microtubule-based neuronal functions, and its contribution to CDD pathology in vivo, remain poorly understood.

Neurons depend on the microtubule cytoskeleton for numerous cellular processes, such as cell division and migration, long-range transport, neurite formation, and synaptogenesis (39–44). Microtubules undergo cycles of rapid growth and disassembly in a process known as dynamic instability (45–47). The dynamic nature of microtubules and their remodelling is necessary for neuronal intracellular transport (48). Microtubule organization and dynamics are crucial for the cargo transport along axons and dendrites driven by molecular motor proteins like kinesins and dynein (49–54). Posttranslational modifications (PTMs) of α- and β-tubulin affect the microtubule architecture, dynamics, and functionality (55, 56). How MAP1S phosphorylation affects PTMs, motor protein function, and cargo transport in neurons and the consequences of these in neuronal function and animal behavior are not known.

To determine the function of MAP1S phosphorylation, we generated a novel MAP1S phosphomutant mouse model (MAP1S S786/812A or MAP1S SA), where the serines are mutated to alanines, which cannot be phosphorylated. We also used a CDKL5-KO mouse model (exon 4 deletion) (57) to compare with our MAP1S phosphomutant mice. Our results reveal that MAP1S phosphorylation at S786/812 is essential for tubulin tyrosination, dynein recruitment to microtubules, dynein-mediated dendritic cargo trafficking in dendrites and synaptogenesis, and cognition. Reexpression of tubulin tyrosine ligase rescues dynein motility, suggesting a critical role for tubulin tyrosination. We conclude that MAP1S phosphorylation leads to synaptic alterations in CDD.

## Results

### MAP1S phosphorylation at S786/812 sites regulates dynein affinity to microtubules and dynein motility.

To study the function of MAP1S phosphorylations, we generated a MAP1S phosphomutant mouse model (MAP1S S786/812A or MAP1S SA) by using CRISPR-Cas9 (58). We note that CDKL5 function can be partially compensated by other kinases with similar substrate specificity (59). Therefore, we expected that the phosphomutant MAP1S SA mouse model could potentially have more enhanced phenotypes when compared with CDKL5 KOs, as these phosphorylations would be completely eliminated. MAP1S S812 phosphorylation (pMAP1S) was significantly reduced in CDKL5 KOs (Supplemental Figure 1, A and B; supplemental material available online with this article; https://doi.org/10.1172/JCI188241DS1), agreeing with our previous results (4). pMAP1S was not detectable in MAP1S SA mice (Supplemental Figure 1, A and B), as expected, since the phosphorylation site is mutated to alanine. Microtubule binding proteins affect a diverse set of microtubule motor protein functions (60). First, we hypothesized that MAP1S phosphorylation and binding to microtubules could influence motor protein association with microtubules. To test this, we performed a cosedimentation assay using brain lysates from CDKL5-knockout (KO) and MAP1S-phosphomutant mice (SA) (Figure 1, A–D). Microtubules were pelleted with the associated proteins after the incubation with the ATP analogue adenylyl-imidodiphosphate (AMP-PNP), which stabilizes motor protein–microtubule interactions (61). Western blotting analysis indicated that the total protein levels of dynein were comparable among brain lysates from CDKL5 KO, MAP1S SA and their respective littermate WT control mice. However, there was a significant reduction of dynein affinity to microtubules in both mutant mice, as shown by dynein intermediate chain (DIC), when brain lysates were treated with AMP-PNP and pelleted by ultracentrifugation (Figure 1, C and D). These data indicate that less dynein is associated with microtubules when MAP1S is not phosphorylated by CDKL5 at Ser786 and 812. In contrast, kinesin-1 (KIF5C) binding to microtubules was not affected in CDKL5-KO (Supplemental Figure 1, C and E) and MAP1S SA mice (Supplemental Figure 1, D and F). By using the same cosedimentation assay, we also showed that MAP1S light chain (LC) binding to microtubules is increased in brain lysates from MAP1S SA cortical lysates when compared with littermate controls (Supplemental Figure 1, G and H). These results are consistent with phosphorylation of MAP1S LC decreasing its affinity for microtubules (4) and extend these findings to in vivo brain samples.

Dynein is a large motor protein that drives transport toward the minus end of microtubules within axons and dendrites (62). Dendritic microtubules are of mixed polarity; therefore, dynein contributes to both retrograde and anterograde transport of cargo in dendrites (63, 64). Since dynein binding to microtubules was impaired in CDKL5 KO and MAP1S SA, we hypothesized that microtubule-based transport in dendrites may be altered in these mice. To test this, we took advantage of an inducible intracellular motility assay to track dynein motility in hippocampal neurons upon rapalog 1 treatment (54). BICD2N-FRB and Pex-mRFP-FKBP plasmid transfection form a chemically inducible system to control organelle positioning inside cells. BICD2N-FRB combines the dynein-recruiting N-terminus of BICD2 with the FRB domain, while PEX-mRFP-FKBP targets FKBP to peroxisomes via peroxisome targeting signal and labels them with mRFP. Upon addition of rapalog, FRB binds FKBP, recruiting dynein to peroxisomes and driving their movement along microtubules. We selected hippocampal primary neurons for live imaging experiments owing to their healthy and relatively less complex cell type content when compared with cortical cultures. CDKL5 is highly active in dendrites, as shown by phosphorylation of its target EB2 (4); therefore, we focused on dendritic transport. We found that dynein motility was impaired, as shown by a reduction in anterograde and retrograde transport, an increase in the number of stationary particles, and increased pause duration in dendrites of CDKL5 KO neurons (Figure 1, E–I). Notably, these defects were even more pronounced in MAP1S SA neurons with reduced anterograde and retrograde transport distance and velocities and increased stationary puncta and pause durations (Figure 1, J–N), as expected from CDKL2’s ability to phosphorylate MAP1S (59). Taken together, these results suggest that when MAP1S phosphorylation is absent or deficient, dynein-microtubule affinity is reduced, impacting dynein-driven transport.

### Loss of MAP1S phosphorylation alters transport of lysosomes and AMPA receptors.

The efficient transport of cargo organelles is crucial for neuronal function. We next aimed to assess whether primary neurons of MAP1S phosphomutant mice have endogenous cargo transport deficits. We transfected primary neurons with Lamp1-RFP, a marker for lysosomes, to visualize their transport along dendrites (Figure 2A). In these videos, we observed the combined net transport of cargo by dynein as well as kinesin motors on dendritic microtubules. Analysis of the time-lapse imaging videos showed significantly reduced travel distances (Figure 2B) and transport velocities (Figure 2C) in the absence of MAP1S S786/812 phosphorylation when compared with WT controls. Lysosome transport in both directions was affected in MAP1S SA neurons. There was a trend for more stationary lysosomes (Figure 2D), and the duration of pauses was significantly increased in the MAP1S SA neurons (Figure 2E). These results are similar to those observed using live imaging of peroxisome transport. Next, we asked whether neurons from MAP1S SA mice have altered transport of synaptic cargoes. For this, we inspected GluA2-containing AMPA receptors by transfecting neurons with pCl-fusionred GluA2(R) (Figure 2F). We observed reduced travel distances (Figure 2G) and reduced retrograde velocity (Figure 2H). These findings were accompanied by an increase in the percentage of stationary AMPA receptor particles (Figure 2I) and longer pauses in neurons from MAP1S phosphomutant mice (Figure 2J). Overall, our data show that when MAP1S S786/812 phosphorylation is compromised, transport of crucial cargoes such as endogenous lysosomes and AMPA receptors is altered in dendrites.

### MAP1S phosphorylation at S786/812 regulates spine morphology and synaptic densities.

Since postsynaptic GluA2 subunit–containing AMPAR transport was significantly reduced in MAP1S SA neurons, we next examined surface GluA2 levels in primary neurons (Figure 3A). Immunofluorescence staining of surface GluA2 receptors showed reduced intensity (Figure 3B) and smaller GluA2 clusters in the secondary dendrites of MAP1S SA neurons (Figure 3C). Additionally, we biochemically analyzed GluA2 levels in total (S1 fraction) and membrane-enriched fractions (P2 fractions) prepared by differential centrifugation (65) (Figure 3D). Again, MAP1S SA lysates showed lower levels of surface GluA2 receptors, (Figure 3F) with total GluA2 levels remaining comparable in both groups (Figure 3E). We next inspected one of the major AMPA receptor subunits, GluA1. Interestingly GluA1 levels in membrane-enriched fractions are also reduced, suggesting that there is an overall deficit in transport and delivery of AMPA receptors to specific compartments in MAP1S SA neurons. These results indicate reduced synaptic AMPA receptors in MAP1S SA mice.

Altered AMPA receptor levels correlate with spine morphology and head size (66). We therefore performed a morphometric analysis of the dendritic spines and density (Figure 3G). We transfected hippocampal neurons from WT and MAP1S SA mice with EGFP and fixed them at DIV18. In MAP1S SA mice, spine density in secondary dendrites was significantly reduced compared with neurons in WT littermate cultures (Figure 3H). Interestingly, the number of mushroom-type spines was affected by the loss of MAP1S phosphorylation and there was a trend for an increased density of immature spines (thin spines) (Figure 3I), suggesting an impairment for spine maturation. Neurons were next stained for the presynaptic protein synaptophysin and the postsynaptic protein PSD-95 (Figure 3G). Colocalized pre- and postsynaptic markers, representing glutamatergic synapses, were significantly reduced in the MAP1S phosphomutant neurons as compared with WT neurons (Figure 3J). The total number of PSD-95 clusters was significantly reduced (Figure 3K) and the total number of synaptophysin clusters was unchanged (Figure 3L), per unit dendritic length in MAP1S SA neurons. The proportion of PSD-95 clusters colocalized with synaptophysin was unchanged (Figure 3M) and the proportion of synaptophysin clusters colocalized with PSD-95 was significantly reduced (Figure 3N) in MAP1S SA neurons. These findings indicate that the reduction in synapses is likely due to reduced PSD-95. Our data therefore suggest that phosphorylation of MAP1S S786/812 is critical in AMPA receptor surface expression, dendritic spine maturation, and excitatory synapse numbers.

### MAP1S phosphorylation at S786/812 regulates microtubule stability and dynamics.

MAP1S directly binds to microtubules and affects their bundling and stability (4, 36, 67). To determine the mechanisms by which MAP1S could be altering transport, we began by examining microtubule stability. For this, we compared the resistance of microtubules from CDKL5-KO and MAP1S SA hippocampal neurons to the depolymerizing drug nocodazole (Supplemental Figure 2, A–D). When neurons were exposed to 30 μM nocodazole for 40 minutes, there was a significant reduction in β-III tubulin intensity in WT neurons, whereas a persistent and higher signal could be observed in CDKL5-KO (Supplemental Figure 2, A and C) and MAP1S SA neurons (Supplemental Figure 2, B and D). Live cell imaging using the fluorescent microtubule +TIP binding protein 3 (EB3) further confirmed that phosphorylation of MAP1S at S786/812 sites affects microtubule dynamics (Figure 4, A–C). In MAP1S SA neurons, we observed that EB3 comets grow over longer distances anterogradely when compared with WT neurons (Figure 4B), mimicking EB3 dynamics in CDKL5-KO mice (68). However, the velocity of each run is comparable in both groups (Figure 4C), meaning that the MAP1S phosphorylation at S786/812 does not affect microtubule polymerization velocity. Lack of MAP1S phosphorylation, and increased MAP1S binding to microtubules, therefore affects microtubule dynamics and stability.

MAP1S binding to microtubules and microtubule stability can alter microtubule posttranslational modifications (PTMs). The reversible addition of tyrosine side chains to the C-terminal tail of tubulins, known as tubulin tyrosination, is a well-established marker for labile, short-lived microtubules (69, 70). Tyrosination is also an important regulator of the initiation of processive dynein (71). We first asked whether microtubule tyrosination levels in hippocampal neurons were altered in CDKL5-KO or MAP1S SA neurons. Immunostaining of hippocampal neurons with a tyrosination-specific antibody revealed significantly decreased levels of tubulin tyrosination in the dendrites of both CDKL5-KO (Figure 4, D and F) and MAP1S SA (Figure 4, E and G) neurons when normalized with costained α-tubulin. On the other hand, the levels of detyrosinated tubulin were increased in both CDKL5-KO (Figure 4, H and J) and MAP1S SA neuronal dendrites (Figure 4, I and K). Western blot analysis using hippocampal lysates derived from WT, CDKL5-KO and MAP1S SA mice also confirmed these results (Supplemental Figure 2, E–H). These data indicate that the microtubule tyrosination/detyrosination cycle is dysregulated in MAP1S SA and CDKL5-KO neurons.

### Tubulin tyrosination reexpression rescues dynein motility defects in MAP1S phosphomutant mice.

As tyrosinated tubulin is necessary for the initiation of dynein transport (71), we hypothesized that this defect may be the primary cause of dynein motility phenotype we observe. To test if the reduction in tubulin tyrosination was the underlying cause of the observed dynein motility deficits, we performed a rescue experiment by expressing human tubulin-tyrosine ligase (hTTL) enzyme in MAP1S SA neurons at DIV5. Primary cultures of hippocampal neurons from MAP1S mice were transfected with either GFP or hTTL-GFP. We first analyzed tubulin tyrosination (Figure 5A) and detyrosination levels (Figure 5B) in neurons transfected with GFP or hTTL-GFP for 24 hours. As expected, hTTL-GFP reexpression normalized tubulin tyrosination levels (Figure 5C) and detyrosination levels in MAP1S SA neurons (Figure 5D). The same neurons were cotransfected with BICD2N-FRB and Pex-mRFP-FKBP and imaged 24 hours later before being fixed and processed for immunofluorescence (Figure 5E). Notably, TTL reexpression for 24 hours ameliorated distance travelled and velocity deficits of the dynein motility in MAP1S SA neurons; yet, the pause duration defect was not reversed (Figure 5, F–I), showing some rescue by TTL reexpression.

Since not only dynein motility but also its affinity to microtubules was impaired in MAP1S SA neurons, we wondered whether either of these phenotypes could be rescued or ameliorated by increasing tubulin tyrosination levels. To test this, we transduced WT cortical neurons with GFP-AAV virus and MAP1S SA cortical neurons with either hTTL-AAV virus or GFP-AAV over 7 days. Cell lysates from these neurons were prepared and processed for cosedimentation assay. Our results confirmed a significant increase in tubulin tyrosination by TTL (Supplemental Figure 3A). We found that dynein affinity to microtubules was reduced in MAP1S SA cortical cultures; however, overexpression of hTTL did not rescue this impairment, despite the increased tubulin tyrosination levels (Supplemental Figure 3, B and C). Overall, these experiments show that increasing tubulin tyrosination in MAP1S SA neurons is sufficient to restore dynein-mediated transport without rescuing its microtubule binding, suggesting that tubulin tyrosination affects dynein function independent of its microtubule binding.

### MAP1S phosphomutant mice display social and motor behavioral deficits.

Several studies have reported a wide range of behavioral deficits in CDKL5 knockout mice, such as altered anxiety, motor performance, and social and cognitive changes (72–74). Since these abnormalities resemble the clinical features of patients with CDD, we wondered whether MAP1S SA mice would show similar behavioral phenotypes. To do that, a cohort of WT and MAP1S SA mice at the age of 3 months was tested with a battery of behavioral tests. To examine whether CDKL5 phosphorylation affects body weight, mice were weighed before the start of the behavioral assessment. Our data showed normal body weights across sexes and genotypes (Supplemental Figure 4, A–C). We started by assessing anxiety-like behavior in our mice. MAP1S SA mice spent less time in the open arms of the elevated plus maze (Supplemental Figure 4, D and E) and less time in the center zone of the open field (Supplemental Figure 4, F and G), suggesting an increase in anxiety in these mutant mice. The activity levels of MAP1S SA mice during 30 minutes of free exploration in the open field were also reduced, prompting us to assess motor performance in these mice (Supplemental Figure 4H). Mice were trained on the rotarod apparatus at a constant speed (4 RPM) and tested 24 hours later at an accelerating speed of 4–40 RPM over 4 minutes (Supplemental Figure 4I). MAP1S SA mice had a poor motor performance shown by the reduced time spent walking on the rotarod (Supplemental Figure 4J). Similar results were observed for the inverted grid test, where MAP1S SA mice spent less time hanging onto the metallic grid (Supplemental Figure 4, K and L). We also tested the hindlimb clasping response in MAP1S SA mice, a behavior that has been reported in mice lacking CDKL5. Unlike CDKL5-KO mice, MAP1S SA mice do not display any hindlimb clasping (Supplemental Figure 4, M and N). In summary, our initial behavioral assessment of MAP1S phosphomutant mice reveals increased anxiety and poor motor performance.

One of the hallmarks of CDD is the aversion to social interaction followed by the development of repetitive behaviors (75, 76). To study this behavior in our MAP1S SA, we used the 3-chamber social test (Supplemental Figure 4O). This test takes advantage of the preference of control mice to explore a social stimulus (an unfamiliar WT mouse) over a nonsocial stimulus (sociability) or novel mouse over a familiar one (social novelty). During the habituation phase, in which the maze lacked both social and nonsocial stimuli, we detected no difference in activity levels between genotypes (Supplemental Figure 4P). In the sociability phase, both genotypes showed an overall preference for the stranger mouse over the object (Supplemental Figure 4Q). Thereafter, in the social novelty phase, WT mice showed a clear preference for the novel mouse when compared with the familiar mouse. This was not the case for MAP1S SA mice, which showed a diminished ability to differentiate between the familiar and the novel mouse (Supplemental Figure 4R) and a reduction in exploratory behavior (Supplemental Figure 4S). Overall, MAP1S phosphomutant mice displayed social deficits and a decreased preference for social novelty.

### MAP1S phosphorylation is important for spatial reference and associative memory.

To assess the role of S786/812 phosphorylation of MAP1S in learning and memory, we employed additional behavioral tests. In a Y-maze spontaneous alternation (SA) test (Figure 6A), MAP1S SA mice performed as well as the control group mice (Figure 6B). These data suggest that MAP1S phosphorylation at the CDKL5 phosphorylation sites is not involved in short-term spatial recognition memory. The overall exploration activity given by the total number of arm entries was comparable across all genotypes (Figure 6C). We next examined context-associative conditioned fear memory in these mice using the contextual fear conditioning paradigm (Figure 6, D and E). On day 1 of the experiment, each mouse received 3 foot shocks and freezing behavior (absence of all movement except respiration) was monitored and used as a readout for associative fear learning and memory in rodents. WT and MAP1S SA displayed equal freezing responses during the training phase of fear conditioning, suggesting normal acquisition of the contextual fear memory (Figure 6F). Twenty-four hours later and upon reexposure to the training context without the foot shocks (context A on day 2), MAP1S SA mice exhibited significantly lower freezing levels, pointing to problems in recalling the context-shock association (Figure 6G). On the other hand, when each mouse was exposed to a different context (context B on day 3), similar freezing levels across genotypes were observed, suggesting a context-specific formation of fear memory (Figure 6H). We next studied extinction of fear memory in the same cohort of mice by reexposing them to the training context A over 4 consecutive days without reinforcement. As expected, WT mice showed decreased context-associated freezing levels, indicating successful weakening of the fear-learned behaviors (Figure 6I). However, that was not the case for MAP1S SA mice, which displayed high levels of freezing behavior over the 4 days. Together, these data indicate that S786/812 phosphorylations of MAP1S is critical for associative memory extinction in mice.

We next assessed the behavior of WT and MAP1S SA mice in the Barnes maze, a test that has been widely used to study spatial learning and memory in rodents. In this test, mice are trained to find the goal box using visual cues to navigate across the maze. Cognitively intact mice spend more time exploring the goal box area or its periphery when the entry to goal box is blocked (Figure 6, J and K). Both genotypes were able to learn the task as shown by decreasing latencies, distance traveled, and errors made to reach the goal box over 4 consecutive days of training (Figure 6, L–N) even though MAP1S SA mice tended to make more errors when trying to reach the goal box (Figure 6N). On the probe test day, MAP1S SA mice spent significantly less time around the goal box (Figure 6O), suggesting that spatial reference memory (short-term) in these mice was impaired compared with the WT control group. Additionally, they were slower (Figure 6P), made more errors (Figure 6Q), and traveled longer distances (Figure 6R) while trying to find the goal box. Together, these results indicate that MAP1S S786/812A mice have impaired spatial reference memory.

## Discussion

In this study, we reveal functional roles of the microtubule-associated protein MAP1S in microtubule cytoskeleton regulation, microtubule-based transport, excitatory synapse formation, and mouse behavior. Using a MAP1S S786/812A phosphomutant mouse model, we provide evidence for the critical role of MAP1S phosphorylation in neuronal synaptic development and organism function. MAP1S S786 and S812 are established CDKL5 phosphorylation sites (4, 23). Loss of CDKL5 results in a significant reduction of MAP1S phosphorylation at S812 to less than 20% of normal levels, although additional kinases are capable of targeting these sites. We have shown that CDKL2 phosphorylates MAP1S S812 in mice (59).

### Transport deficits and excitatory synapses in MAP1S SA mice.

Using 2 mouse models, CDKL5 KO and MAP1S SA, we showed that MAP1S phosphorylation hampers dynein affinity to microtubules and motor protein processivity. By recruiting dynein to peroxisomes using a rapalog-BicD system, we revealed a clear reduction in dynein-mediated transport in these mouse models. In addition, using RFP-tagged Lamp1 or PfusionRed-tagged GluA2, we were able to show that cargo transport was affected in dendrites. These results are in line with previous data showing a significant reduction in anterograde transport of BDNF/TrkB in the dendrites of CDKL5-depleted neurons (4).

Reduced transport of synaptic cargo such as GluA2-containing AMPA receptors to the postsynapse may account for the abnormal dendritic spine morphology we observed in MAP1S SA mice. Spine morphology phenotypes were also observed in CDKL5-KO mice (77, 78). The trafficking of cargo along the dendrites is mainly carried out by dynein and kinesin motors, and the net movement is determined by the combined activity of both motor proteins for both directions. Dynein is a minus-end–directed motor; due to the mixed polarity of microtubules in dendrites, dynein contributes to both retrograde and anterograde motility of cargo in dendrites (42, 64). Based on our data, we propose that dynein-driven transport is modulated by MAP1S phosphorylation levels, which contribute to the impairments of cargo trafficking in both anterograde and retrograde directions in dendrites.

### Microtubule tyrosination as a critical modification for dynein-mediated transport.

In the absence of CDKL5 or when MAP1S is not phosphorylated, overstabilization of microtubules by MAP1S occurs and tubulin tyrosination is negatively regulated. In support of MAP1S’s role in microtubule stability, COS-7 cells overexpressing MAP1S light chain have very stable microtubules, as shown by increased resistance to depolymerization upon colchicine treatment (36). Additionally, our findings on Eb3 dynamics in MAP1S phosphomutant neurons recapitulate the altered microtubule dynamics reported in CDKL5-KO neurons. In the absence of CDKL5, MAP1S has a higher affinity to microtubules and renders them less dynamic, as seen by longer EB3-GFP comets, but this could be rescued by MAP1S shRNA expression (4).

Among the microtubule PTMs, the tubulin tyrosination/detyrosination cycle has been implicated in the regulation of microtubule stability and dynamics. Briefly, when the C-terminal tyrosine of the α-tubulin is removed by a carboxypeptidase, detyrosinated microtubules are formed. The enzymatic readdition of the tyrosine by tubulin-tyrosine ligase (TTL) leads to tyrosinated microtubules, which are more labile and dynamic (79, 80). Deficits in posttranslational detyrosination/tyrosination levels have been associated with impaired axonal transport, spatial learning, synaptic plasticity, and loss of synapses (81, 82).

TTL expression normalized tyrosinated tubulin levels in neurons and ameliorated some of the dynein motility impairments, indicating that reduced tubulin tyrosination underlies the dynein motility defects in MAP1S SA mice. Interestingly, hTTL expression did not affect the binding of dynein on microtubules in cosedimentation experiments. These data differentiate the effect of tubulin tyrosination on dynein binding from dynein motility. It is possible that MAP1S affects the recruitment of TTL or tubulin detyrosinating enzymes (vasohibins) to microtubules (70). For example, MAP7 association with microtubules inhibits tubulin detyrosination by sterically blocking vasohibin 1 recruitment, and, at the same time, protects acetylated microtubules and enhances kinesin-1 binding (83, 84). We also cannot rule out the steric interference caused by increased MAP1S binding to microtubules that could render dynein binding to microtubules less efficient. While dynein binding to microtubules could be impaired due to MAP1S excessive binding to microtubules, rescuing dynein-mediated transport by increasing tubulin tyrosination did not require a rescue of dynein binding. This indicates that tyrosination does not affect dynein binding directly but acts as a regulatory mechanism for productive transport. These results agree with prior reconstitution experiments showing dynein can still bind to microtubules without tyrosination, but it fails to initiate processive, dynactin-driven transport (52, 71).

Several studies have reported that dynein and adaptor proteins like p50^glued^ and the microtubule-plus–end tracking protein CLIP-170, which enhance recruitment of dynein, bind preferentially to microtubules enriched in tyrosinated tubulin (52, 60). Importantly, CLIP-170 binding to microtubules has been reported to be deregulated in CDKL5-KO mice. Treatment with PME (pregnenolone-methyl-ether), a compound that promotes the proper localization of CLIP-170 to microtubules, was found to improve CDD-associated neuronal deficits like spine maturation and GluA2-containing AMPA receptor levels at the postsynapse (85). These studies show that microtubule-based transport defects are a key component of CDKL5 deficiency during neuronal development. While dynein motility and synaptic analyses were performed in cultured neurons, tyrosination and dynein-binding analyses were conducted in both cultured neurons and in vivo models, strengthening our conclusions.

### MAP1S phosphorylation in learning and CDD pathology.

CDD is a complex clinical condition characterized by early onset seizures, impaired locomotion, and cognitive deficits accompanied by psychiatric comorbidities (86). However, the subcellular mechanisms underlying these abnormal behaviors remain to be elucidated. In this study, we performed a comprehensive behavior analysis of MAP1S phosphomutant mice and their WT littermates. MAP1S-KO mice have histological phenotypes in the heart associated with autophagy impairment (87). MAP1S SA mutation specifically enhances microtubule binding and would not be expected to have a null phenotype. MAP1S SA mice did not have weight or lifespan changes and were overall healthy; therefore, we do not expect MAP1S SA to have significant autophagy impairment. Nevertheless, we cannot rule out the possibility that some of the behavioral phenotypes we describe in MAP1S SA may be due to additional molecular roles of MAP1S.

Our analysis revealed similarities as well as differences in MAP1S SA behavioral phenotypes when compared with those previously reported in CDKL5-mutant mice. While MAP1S SA mice showed increased anxiety-like behavior in elevated plus maze and open field test, CDKL5-null (74) and CDKL5-STOP mice (genetically reversible null mice) (88) showed reduced anxiety-like behavior, while CDKL5 nulls were also reported to have unchanged anxiety (89) or increased anxiety (72). MAP1S SA mice did not show any significant differences in working memory, as indicated in the Y-maze, which is known to be impaired in CDKL5-KO (72, 73), CDKL5-STOP (88), and CDKL5-R59X transgenic mice (90). Finally, MAP1S SA mice do not present hindlimb clasping, a phenotype consistently observed in CDKL5 KOs and they showed hypoactivity in open field test, whereas CDKL5 KOs present with hyperactivity (74, 88, 89). These differences can be explained by the contribution of several other CDKL5 substrates, such as EB2 and Cav2.3 (4, 91), to the behavioral phenotypes in CDKL5-KO mice.

MAP1S SA behavioral phenotypes also showed overlap with CDKL5-mutant mice. While we observed sociability deficits that mirror CDKL5-KO mice (88, 89, 92). Fear memory recall and extinction as well as spatial memory were impaired in MAP1S SA mice, which are also observed in CDKL5-KO mice (72, 74, 93, 94). Thus, our data support a role of MAP1S phosphorylation at S786/812 in memory formation.

At the cellular level, we demonstrated that MAP1S phosphorylation affects spine morphology, a cellular substrate of memory. We postulate that the effect of MAP1S on memory is due to MAP1S’s roles on microtubule stability and tubulin posttranslational modifications, which impinges on cargo transport to synapses. It is well established that the dendritic spines are sites for the majority of excitatory synapses in the mammalian brain, and the spine head enlargement via insertion of AMPA receptors during plasticity is widely accepted to be crucial for learning and memory. Mushroom spines, with larger spine heads, harbor more stable synapses and are less likely to turn over, implicating them in memory (95).

Previous work shows that the dynamic microtubules entering spines regulate synaptic plasticity (43). In addition, deletion of fibroblast growth factor 13 (FGF13), a microtubule-stabilizing protein, leads to alterations in learning and memory (96). Another important regulator of microtubule stability is the microtubule-severing enzyme spastin. Neurons lacking spastin display higher levels of polyglutamylated microtubules, reduced microtubule dynamics accompanied by cargo transport and delivery impairments, reduced numbers of excitatory synapses, and learning and memory deficits (97). Interestingly, tubulin tyrosination regulates dendritic spine density, microtubule dynamics, and cognition, and is downregulated in patients with Alzheimer’s disease (82). Our study suggests that altering normal microtubule architecture disrupts the delivery of different intracellular cargoes, thereby reducing mature spine and synapse numbers. Therefore, our results reveal MAP1S function as a critical part of microtubule regulation in neuronal dendrites, leading to altered AMPA receptor insertions, spine morphology, and learning and memory.

In summary, we find that loss of MAP1S phosphorylation is pathological because it overstabilizes microtubules and reduces overall tubulin tyrosination, which, in turn, leads to reduced dynein-mediated transport and subsequent synaptic and cognitive deficits. Our results reveal a distinct mechanism by which CDKL5 affects neuronal development.

## Methods

### Sex as a biological variable.

Both male and female mice were used in our study. Sex was not considered as a biological variable for analysis conducted in primary neuronal cultures or ex vivo experiments on brain sections and brain homogenates. For behavior experiments where sex is considered as a variable, male and female cohorts were analyzed separately. Analysis was also shown when both sexes are combined.

### Mice.

All animal procedures were performed in strict accordance with the regulations of the Animal (Scientific Procedures) Act 1986 and approved by The Francis Crick Institute ethical committee. Behavioral experiments were conducted on male and female mice. CDKL5 knockout mice were a kind gift from Dr. Cornelius Gross, EMBL Rome, Rome, Italy (57).

MAP1S phosphomutant (SA) mouse was generated by GEMS at the Francis Crick Institute. MAP1S point mutation of S786A and S812A was created using CRISPR Cas9 directed mutagenesis. Two guides were used: sgRNA-1 TOP 5’: AGGG GGCAGCTACAGTTGCAGCTC; BOTTOM 5’: AAAC GAGCTGCAACTGTAGCTGCC; sgRNA-2 TOP 5’: AGGG GGCTCACTGCGGGCGCTGAG; BOTTOM 5’ AAAC CTCAGCGCCCGCAGTGAGCC; 200bp HDR ssODN: CTCAACACCACCAATCCCAGCCGTAGCCGCAAGGCCCCTGCAAGGCCTAGCgCTGCCTCTGCCACCCCtAGAGCTGCAACTGTAGCTGCCAAGACAAAGGGCCCCGCGGGcGAtCGGAACCGGCCACTCgcCGCCCGCAGTGAGCCTGCAGACAGGCCAGGCCGTGTGCCCCTTACCAGGAAGCCCTCAGTCCCTAAGAC; MAP1S, Exon 5: Amino acid S786A — TCT to gCT and amino acid S812A — AGC to gcC. PAM silent mutation from TTG to TaG (for Guide Sequence 1). Guide Sequence 2 PAM does not require a mutation due to the S812A change being in the seeding region of the guide. Restriction site PvuI introduced as a SM for genotyping.

CRISPR guides have been carefully selected to have at least 3 mismatches with off-target sites. Among these sites, the vast majority of which are intronic or intergenic, leaving only 1 exonic site which is on chromosome 2. None of these 3 mismatch sites are on the same chromosome as the Map1s gene (chromosome 8). We have kept our colony as heterozygotes and crossed periodically with WT mice (Jackson C57BL6). These mice have been crossed for multiple generations before the start of our experiments, which lasted multiple years and further generations. Therefore, a co-segregation of an off-site mutation is exceedingly unlikely.

### DNA constructs.

The following plasmids were used for transfection: EB3-tdTomato was a gift from Erik Dent, University of Wisconsin-Madison, Madison, WI, USA (Addgene plasmid #50708), pTRIP-CAG-hTTL-GFP and pTRIP-CAG-GFP were a gift from Carsten Janke, Institut Curie Orsay, Orsay, France. HA-BICD2N-FRB-PCI-NEO and GW1-PEX-mRFP-FKBP were gifts from Lukas Kapitein, Utrecht University, Utrecht, Netherlands. Lamp1-RFP and pCl-fusionred-GluA2(R) were a gift from Daniel Choquet, CNRS / Université de Bordeaux, Bordeaux Cedex, France. All plasmids were confirmed by DNA sequencing.

### Adeno-associated virus production.

The AAV was produced as previously described in Challis et al. Briefly, HEK-293T cells (Francis Crick Institute) cultured in DMEM-10% FBS with 1x Pen/Strep at 50-70% confluency were triple transfected with pHelper:pAAV2/1 (Agilent technologies): transfer plasmid in a 1:1 or 3:1 molar ratio using PEI-MAX (per 15 cm dish: 1 ml Optimem,12 μg pHelper, 10 μg pAAV2/1, and 6 μg transfer plasmid -add 70 μl 1 mg/ml PEI-MAX). Six hours later the media was replaced with fresh media and the cells kept in the incubator at 37°C. The AAVs were harvested 72h-post transfection using 8% PEG8000-82mM NaCl precipitation and chloroform extraction followed by PBS exchange in concentration columns. For the viral production, 3 × 10 cm dishes were used, resulting in 50 μl of purified AAV vector with a titer of 6 × 10^13^ vg/ml. Purified AAVs were titered using the method described in Aurnhammer et al. on a Quant3 (Perkin Elmer) using PowerUp SYBRgreen master mix. pAAV2/1 was a gift from James M. Wilson, UPenn, Philadelphia, USA (Addgene plasmid# 112862).

### Microtubule binding assay.

For co-sedimentation assays, we used a modified version of a protocol previously described (61). In brief, cortices were harvested from P20 mice and homogenized in microtubule-stabilizing buffer (1 mM EGTA/100 mM PIPES,5 pH 6.8/ 1 mM MgSO_4_) containing protease inhibitors (10 μM PMSF/5 μg/ml E64). The cortical homogenates containing the crude tubulin were centrifuged at 50,000 × *g* (Beckman Coulter, Optima Max-XP) for 30 minutes at 4°C to yield a supernatant, whereas the pellet was discarded. Afterwards, the supernatant was supplemented with 1 mM GTP, 20% glycerol, 20 μM Taxol, 1 mM AMP-PNP (pH 6.8) and incubated at 37°C for 35 minutes, followed by a centrifugation step at 150,000 × *g* for 40 minutes at 37°C. The pellet containing polymerized microtubules and co-pelleted microtubule-associated proteins was resuspended in 8 M urea for 30 minutes at RT and processed for western blotting analysis.

### Primary neuronal cultures and live imaging of neurons.

For CDKL5 WT and CDKL5 knockout littermate cultures, we crossed CDKL5 heterozygous females with WT male mice and we used the resulting male embryos; each embryo was cultured separately. For MAP1S SA primary neuronal cultures, heterozygous females were crossed with heterozygous males and only homozygous knockouts and WT embryos were used. We used both sexes: we combined homozygous knockout males and females (MAP1S SA condition), we combined WT males and females (WT condition). Primary hippocampal neurons were prepared as described and cultured on glass bottom dishes. Neurons were transfected using Lipofectamine® 2000 reagent protocol and imaged 24 hours later. Time-lapse live-cell imaging was performed on a Nikon CSU-W1 Ti2 Confocal microscope with a Dual Prime 95B camera with a 100× (oil) objective coupled to an incubator chamber, where hippocampal neurons were maintained in conditioned medium and kept at 37°C and 5% CO_2_. For the dynein motility experiments, DIV6 neurons were co-transfected with HA-BICD2N-FRB-PCI-NEO, GW1-PEX-mRFP-FKBP and eGFP-C1 and imaged at 7 frames per second for 2 minutes. Prior to live imaging, the neurons in conditioning medium (1 ml total volume) were incubated with 8 μl of 0.1 mM rapalog1 (AP21967, Ariad Pharmaceuticals) for 5 minutes. (Supplemental Figure 1I) For the rescue experiments, in addition to HA-BICD2N-FRB-PCI-NEO, GW1-PEX-mRFP-FKBP plasmids, neurons were co-transfected with pTRIP-CAG-hTTL-eGFP or pTRIP-CAG-eGFP for 24 hours. For GluA2 transport studies, neurons at DIV5 were transfected with pCl-fusionred-GluA2(R) and imaged at 5 frames per second for one minute. To visualize EB3 comets, neurons at DIV4 were transfected with the microtubule-plus-end marker EB3-tdTomato and imaged at 1 frame per second for 3 minutes. Flat stretches of dendrites with low expression levels of EB3-tdTomato were selected for the analysis. For lysosome transport, neurons at DIV6 were transfected with Lamp1-RFP and imaged at 5 frames per second for 2 minutes. For the final analysis, captured time-lapse videos were exported as TIF images. Anterograde and retrograde movement of dynein, GluA2 single particle mobility, lysosomes as well as EB3-tdTomato comet growth of sequential images within secondary dendrites identified based on their morphology were quantified semi-manually using KymoAnalyzer plugin (98) in Fiji (https://fiji.sc/). Background correction was achieved through subtraction of average intensity using Fiji software. Kymographs of 30 μm (Dynein, GluA2, Lysosomes and EB3 comets) dendritic segments were generated using the Multiple Kymograph plugin for Fiji.

### Nocodazole susceptibility assay.

Cultured wild-type and MAP1S phosphomutant hippocampal neurons were treated with 30 μM nocodazole (Sigma) in DMSO or with DMSO alone for 40 minutes, permeabilized in PHEM buffer (60 mM PIPES, 25 mM HEPES, 10 mM EGTA and 2 mM MgCl_2_, pH 6.9) with 0.05% Triton^®^-X 100, and 10 μM taxol and fixed in PHEM buffer with 4% PFA. The microtubule cytoskeleton was analyzed using a beta-III-tubulin immunofluorescence.

### Behavioral analysis.

The composition of the 3-month behavior cohort consisted of the following: WT males, *n =* 10; WT females = 10; MAP1S SA males, *n =* 11; MAP1S SA females, *n =* 10.

### Y-maze spontaneous alternation test.

Working memory was measured on an apparatus consisting of a Y-shaped maze with three arms made of white plastic opaque walls (34 × 9 × 14 cm) oriented in a 120° angle. For testing, the mouse was randomly placed into one arm, facing the center and allowed to freely explore the maze for 10 minutes with dim lighting (10 lux). An arm entry or exit was defined as when the mouse moved beyond the boundary of the center zone with all four paws. An actual alternation was counted when a mouse explored three different arms on overlapping triplet sets (e.g.: 1, 2, 3 or 2, 1, 3 or 3, 2, 1). The percentage of spontaneous alternation was calculated as the ratio of actual alternations to the maximum number of possible alternations (calculated as the total number of arm entries minus 2). Each trial was recorded using a video-tracking system (Ethovision XT 15).

### Barnes-maze test.

The Barnes maze consisted of a white, horizontal circular platform (96 cm in diameter, elevated 90 cm above the floor) containing 20 evenly spaced holes. Around the maze, four signs were hung on the walls of the room to serve as navigation cues. The maze was kept in a brightly illuminated room (635 lux) and underneath the target hole, a dark escape box (20 × 11 × 7 cm) was fixed. Each mouse was placed in the center of the maze and allowed to freely explore it for 3 minutes. In case the mouse did not find the escape box, the experimenter will gently guide the mouse to the target hole. Each mouse was tested in two trials per day for four consecutive days (training phase) with an inter-trial time of at least 20 minutes and the location of the escape box was kept constant. To assess learning, a 3-minute probe test was performed with the escape box being removed, and the entry blocked before the mouse was tested. Distance to first: distance traveled from the center of the arena to the target hole (center-point detection); latency to first: time to reach the target hole from the center of the arena (center-point detection); errors to first are defined as checking any hole before reaching the escape box (nose-point detection).

### Contextual fear conditioning.

Contextual fear conditioning is a widely used paradigm to study learning and fear memory after aversive stimulus, typically a mild foot shock is delivered. On day 1, the mouse was placed in the conditioning shock chamber (context A) consisting of a box with opaque walls (27 × 27 × 35 cm) and equipped with digital NIR video fear conditioning system (Ugo Basile, Italy) coupled to a computer. Each trial lasted 8 minutes and every 2 minutes a foot shock (unconditioned stimulus, US) was given to the mouse (0.25 mA, duration: 1 s). Contextual visual cues were added to the context A to assist with the association context-foot shock: vanilla sent, a visual sign on one of the walls and the light was kept on (100 lux). On day 2, To examine the conditioned response to the context, mice were placed back into the same context but without receiving foot shocks for 5 minutes. On day 3, mice were introduced to a different context for 5 minutes (context B), where the grid floor was covered with a plastic insert, no vanilla sent, no illumination and no visual cue was added. From day 4 to day 7, extinction of freezing behavior in mice was assessed. Fear extinction is expected to occur when context A no longer predicts unconditioned stimuli, which is described as a new form of learning). The freezing behavior of each mouse was videotaped and analyzed using Ethovision XT15 (Noldus, Leesburg, Virginia, USA).

### Statistics.

All quantifications throughout this study are based on at least 2–3 independent experiments. Immunoblotting was performed using standard methodologies and the quantification of optical densities of respective proteins was done using ImageJ software (NIH) or Image Studio Lite (LI-COR). Signal intensities were then normalized, as compared with loading control signal intensities and the mean of control data points was set to 100% maintaining the variance.

Maximum projection of z-stacks of confocal images (z-step: 0.5 μm) was used for morphometric analysis of fixed hippocampal neurons using Metamorph software (Universal imaging). The intensity signal for each channel of the merged TIF images was determined by applying the ‘‘Integrated Morphometry Analysis’’ function and image thresholds, brightness was adjusted using the ‘‘Inclusive thresholding state’’ function.

Time-lapse images of live neurons were processed using FIJI/ImageJ. One dendrite per neuron was manually traced using the segmented line tool with a 5-pixel width, and the kymograph was generated from this ROI using the MultipleKymograph plugin. The kymographs were then analyzed using the KymoAnalyzer software. For each individual track, the program calculated the net displacement, net velocity, pause duration, and the percentage of particles identified as stationary or moving in the retrograde or anterograde direction. A threshold value of 350 nm was used to discriminate between stationary and mobile particles. 0.1 μm/second was the cutoff value set to identify a pause.

All statistical analysis and graphical representation were conducted using Graphpad Prism (version 10, Graphpad Software, Inc, San Diego, California, USA) or SPSS Statistics (version 19.0, Armonk, New York, USA; IBM Corp). Behavioral data were first analyzed for deviation from normal normality using “Shapiro-Wilk’s W’’ statistic and the homogeneity of variances using Levene’s test. Statistical significance between groups was determined by ANOVA with independent factors of genotype and sex or 2-tailed unpaired Student′s *t* test, as indicated in the figure legends. When no statistical evidence for genotype × sex interactions or main effects were reported, data from both sexes were plotted together. During each experiment, the experimenter was blind to the genotype or treatment. Comparisons were considered statistically significant when *P < 0.05. Data are presented as mean ± SEM.

### Study approval.

All animal procedures were performed in strict accordance with the regulations of the Animal (Scientific Procedures) Act 1986 and approved by The Francis Crick Institute ethical committee.

### Data availability.

All data generated in this study can be found in the Supporting Data Values file.

## Manuscript information

### Author contributions.

ATL, Conceptualization, Formal analysis, Investigation, Software, Data curation, Visualization, Writing; OJ, Formal analysis, Investigation, Visualization; SC, Formal analysis, Investigation; SKU, Conceptualization, Data curation, Investigation, Funding acquisition, Methodology, Project administration, Supervision, Validation, Writing.

### AI disclosure.

The authors declare that no AI tools were used in the original research for or writing of this article.

### Conflict of interest.

The authors have declared that no conflict of interest exists.

### Funding.

This study was supported by the following:

- Cancer Research UK (CC2037), the UK Medical Research Council (CC2037) and the Wellcome Trust (CC2037) (to The Francis Crick Institute).
- LouLou Foundation Project Grant (11015) (to SKU).

## Supplementary Material

Supplemental data

Unedited blot and gel images

Supporting data values

## Figures and Tables

**Figure 1:**
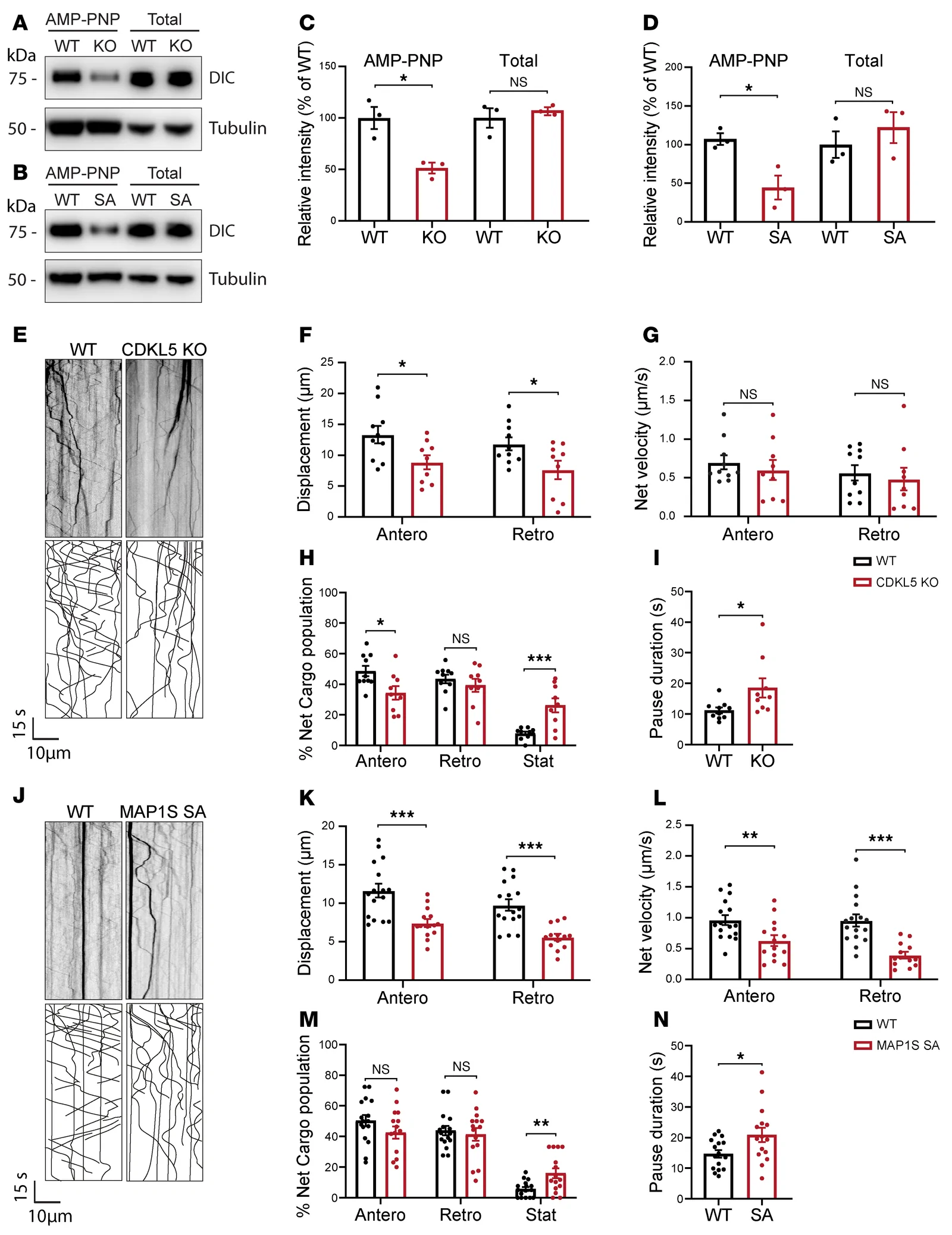
Effects of MAP1S S786/812 on binding of dynein to microtubules and dynein motility. (**A** and **B**) Crude microtubules were prepared from cortical homogenates of WT, CDKL5-KO and MAP1S phosphomutants in the presence of AMP-PNP. (**C** and **D**) Total and pelleted DIC (Dynein intermediate chain) were detected by Western blot analysis and normalized against tubulin. Signal intensities were quantified and are represented as percentage of the mutant signal relative to WT signal. WT, *n =* 3, CDKL5-KO, *n =* 3, MAP1S SA, *n =* 3, mice. (**E**–**N**) Live imaging of dynein-pex transport in dendrites from WT and CDKL5-KO and MAP1S SA hippocampal neurons at DIV7. (**E** and **J**) Kymographs: horizontal scale bar: 10 µm, vertical scale bar: 15 seconds. (**F** and **K**) Displacement. (**G** and **L**) Net velocity. (**H** and **M**) Percentage of net cargo populations. (**I** and **N**) Pause duration. Images were taken at 7 frames per second. WT, *n =* 10–16 cells, CDKL5-KO, *n =* 9 cells, MAP1S SA, *n =* 15 cells from 3 independent experiments. Two-tailed Student’s *t* test was used to assess statistical significance. *P < 0.05, **P < 0.01 ***P < 0.001. Data are represented as mean ± SEM.

**Figure 2:**
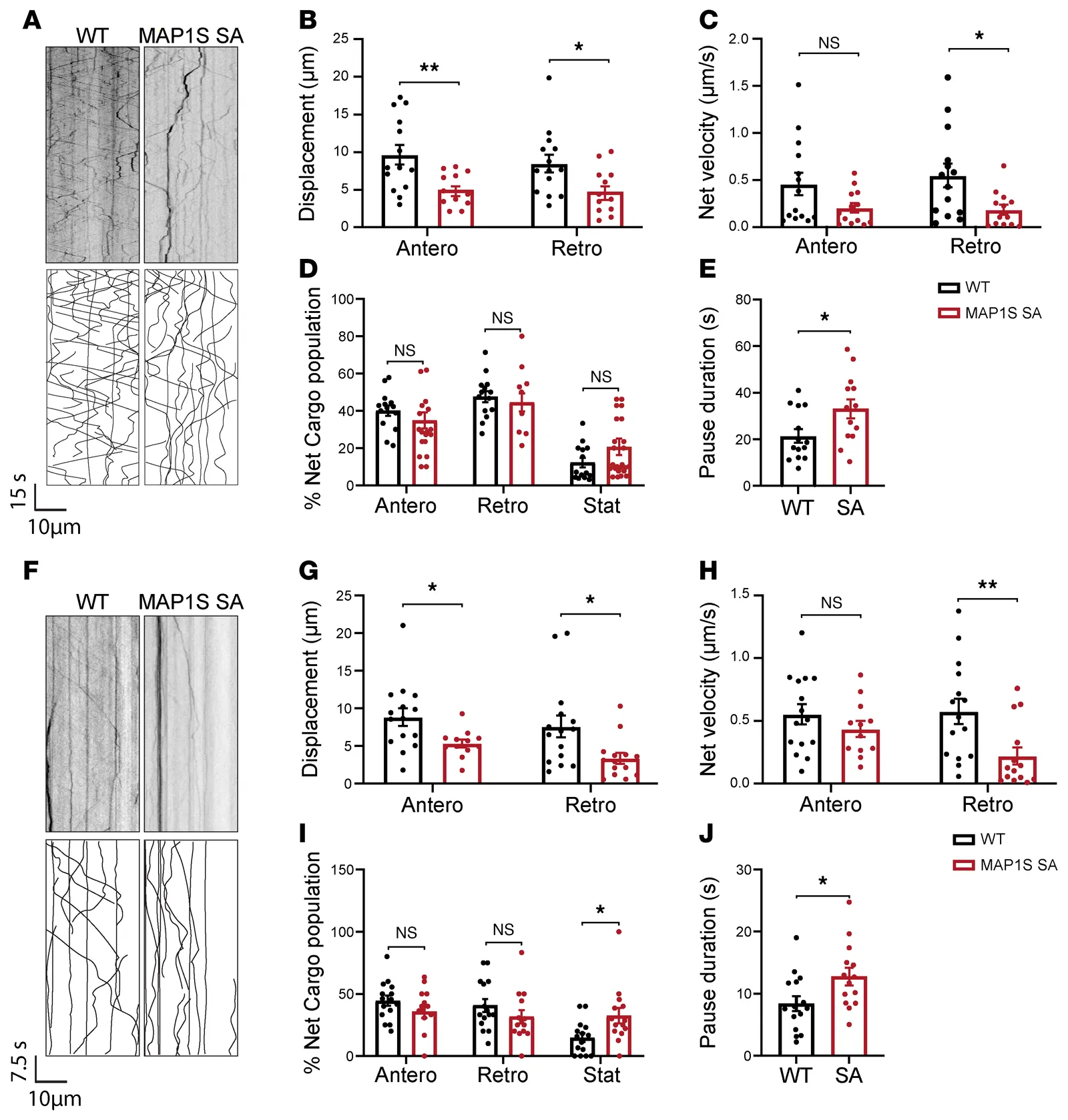
Cargo transport is impaired in MAP1S SA neurons. (**A**–**E**) Live imaging of lysosome movement in dendrites from WT and MAP1S SA hippocampal neurons at DIV7. (**A**) Kymographs from neurons expressing LAMP1-RFP. Kymograph: horizontal scale bar: 10 µm, vertical scale bar: 15 seconds. (**B**) Displacement. (**C**) Net velocity. (**D**) Percentage of net cargo population. (**E**) Pause duration. WT, *n =* 14 cells, MAP1S SA, *n =* 13 cells from 3 independent experiments. (**F**–**J**) GluA2 transport in dendrites from WT and MAP1S SA hippocampal neurons at DIV7. Kymograph: horizontal scale bar: 10 µm, vertical scale bar: 7.5 seconds. (**G**) Displacement. (**H**) Net velocity. (**I**) Percentage of cargo populations. (**J**) Pause duration. Images were taken at 5 frames per second. WT, *n =* 15, MAP1S SA, *n =* 14 from 3 independent experiments. Two-tailed Student’s *t* test was used to assess statistical significance. *P < 0.05, **P < 0.01, ***P < 0.001. Data are represented as mean ± SEM.

**Figure 3:**
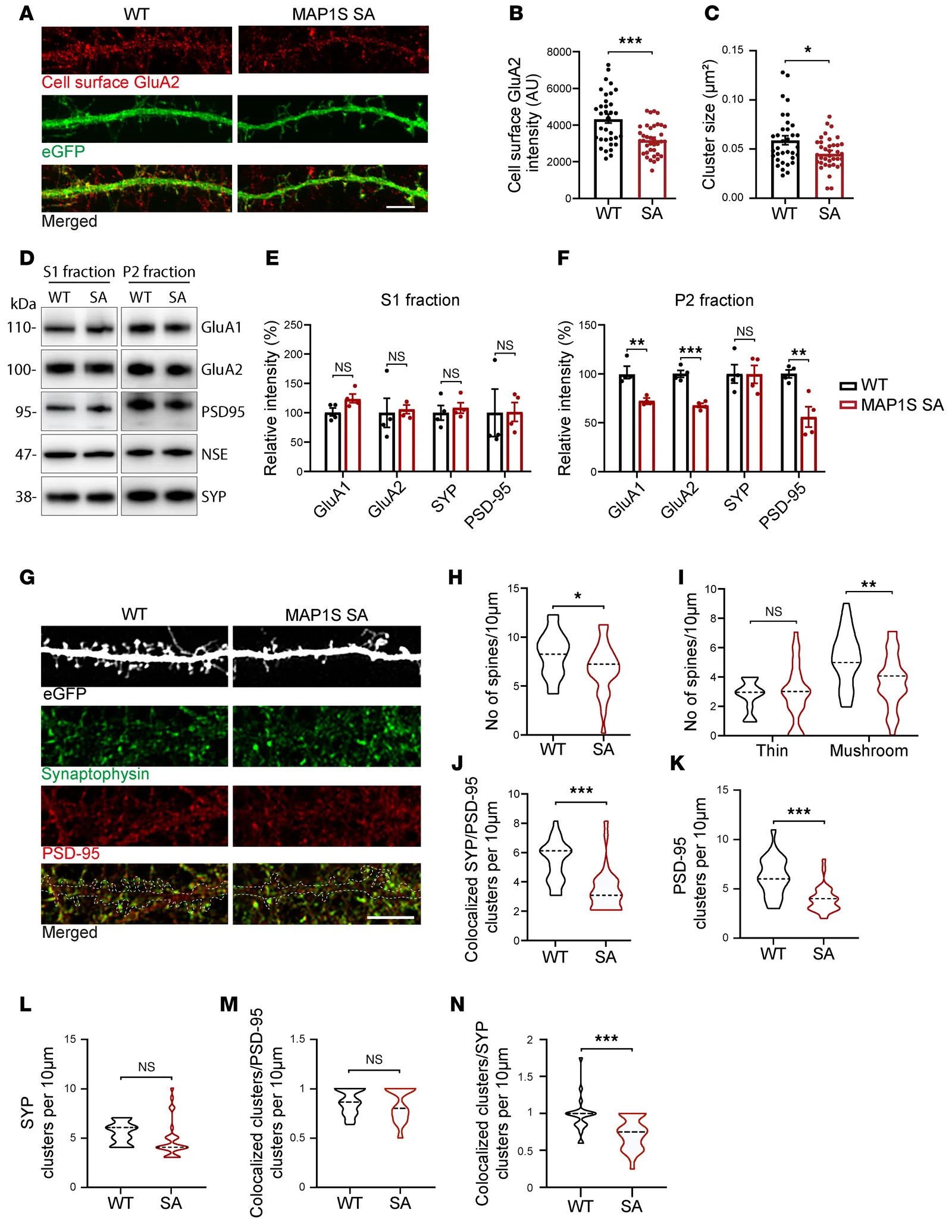
MAP1S phosphorylation regulates spine morphology and synapse density. (**A**) GluA2-AMPAR cell surface staining on dendrites of cultured hippocampal neurons from WT and MAP1S SA mice at DIV14. Cell surface (red) expression in dendrites, GFP as a volume marker. Scale: 5 μm. (**B** and **C**) Quantification of cell surface GluA2 intensity and GluA2 cluster size, respectively. WT, *n =* 16 cells, MAP1S SA, *n =* 16 cells from 3 independent experiments. (**D**–**F**) Western blot analysis of GluA1, GluA2, PSD-95, and synaptophysin levels normalized against neuron-specific enolase (NSE) in S1 fraction (total levels) and P2 fraction (crude synaptosomal fraction) from WT and MAP1S SA mice. *n =* 4 mice per genotype. (**G**) Representative images of eGFP-transfected neurons at DIV18 stained for SYP and PSD-95 to visualize excitatory synaptic contacts. Scale bar: 5 μm. (**H**) Quantification of total number of spines per 10 μm of dendrite length. (**I**) Quantification of spine types per 10 μm dendrite length. WT, *n =* 24 cells, MAP1S SA, *n =* 29 cells from 3 independent experiments. (**J**) Quantification of excitatory synapses per 10 μm dendrite length at DIV18. (**K**) Number of PSD-95 clusters per 10 μm dendrite length. (**L**) Number of synaptophysin (SYP) clusters per 10 μm dendrite length. Ratios of excitatory synapses (colocalized clusters) to total number of PSD-95 clusters (**M**) or to total number of synaptophysin (**N**). WT, *n =* 24 cells, MAP1S SA, *n =* 29 cells from 3 independent experiments. Two-tailed Student’s *t* test was used to assess statistical significance. *P < 0.05, **P < 0.01, ***P < 0.001. Data are represented as mean ± SEM.

**Figure 4:**
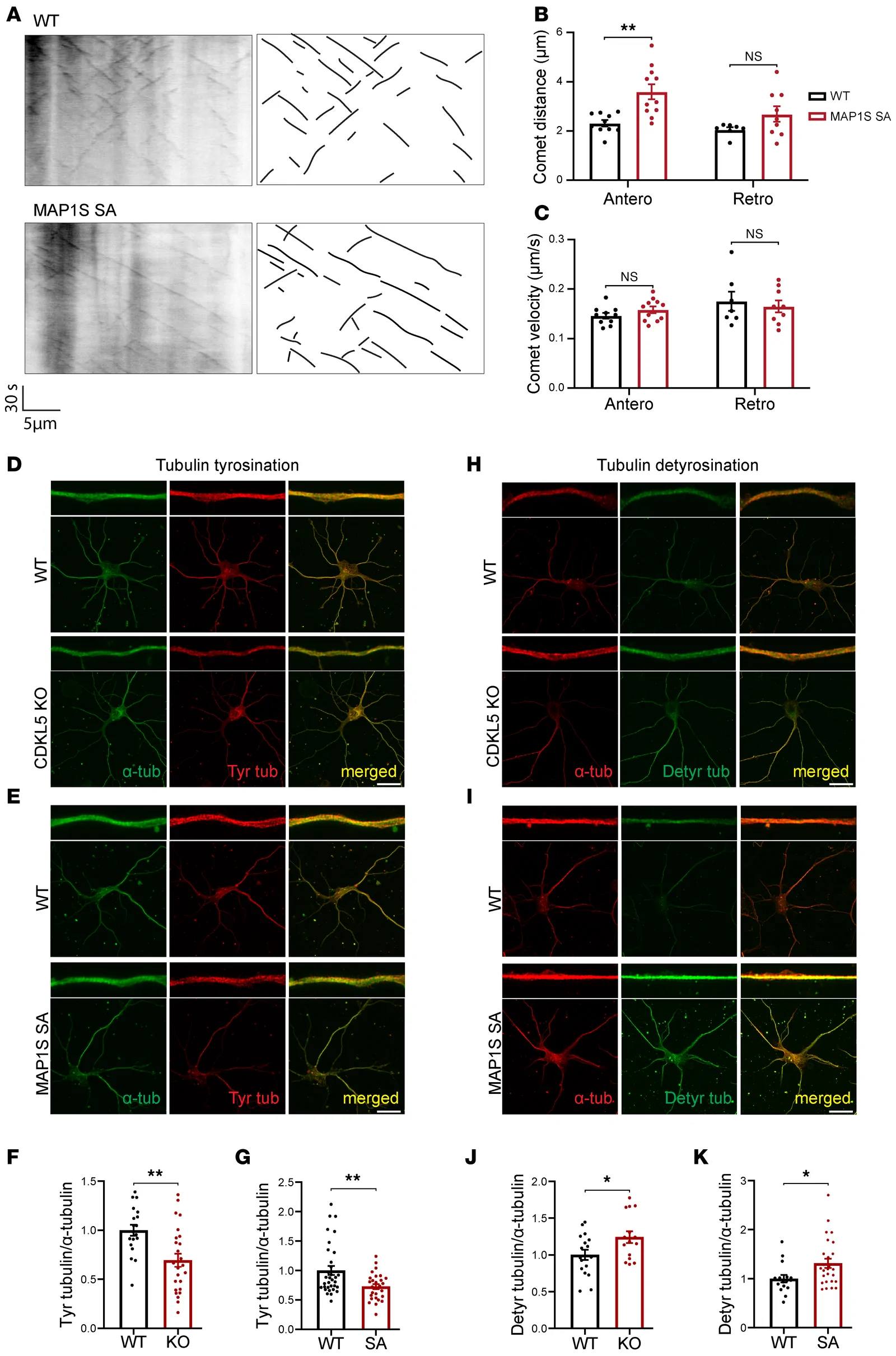
Regulation of microtubule stability and dynamics in MAP1S S786/812 and in CDKL5-KO mice. (**A**) Microtubule dynamics in WT and MAP1S SA neurons. Representative kymographs of EB3-tdTomato acquired in dendrites from WT and MAP1S SA neurons at DIV5. Quantification of anterograde comet distance, retrograde comet distance (**B**), anterograde comet velocity, retrograde comet velocity (**C**). WT, *n =* 10 cells, MAP1S SA, *n =* 11 cells from 2 independent experiments. Coimmunostaining of α-tubulin with tyrosinated tubulin (**D** and **E**) and detyrosinated tubulin (**H** and **I**) in WT, CDKL5-KO and MAP1S SA neurons at DIV5. (**F** and **G**) Quantification of immunofluorescence signal in **D** and **E**. (**J** and **K**) Quantification of immunofluorescence signal in **H** and **I**. Scale bar: 20 μm. WT, *n =* 16–33 cells, CDKL5 KO, *n =* 15–25 cells, MAP1S SA, *n =* 26–28 cells from 3 independent experiments. Two-tailed Student’s *t* test was used to assess statistical significance. *P < 0.05. Data are represented as mean ± SEM.

**Figure 5:**
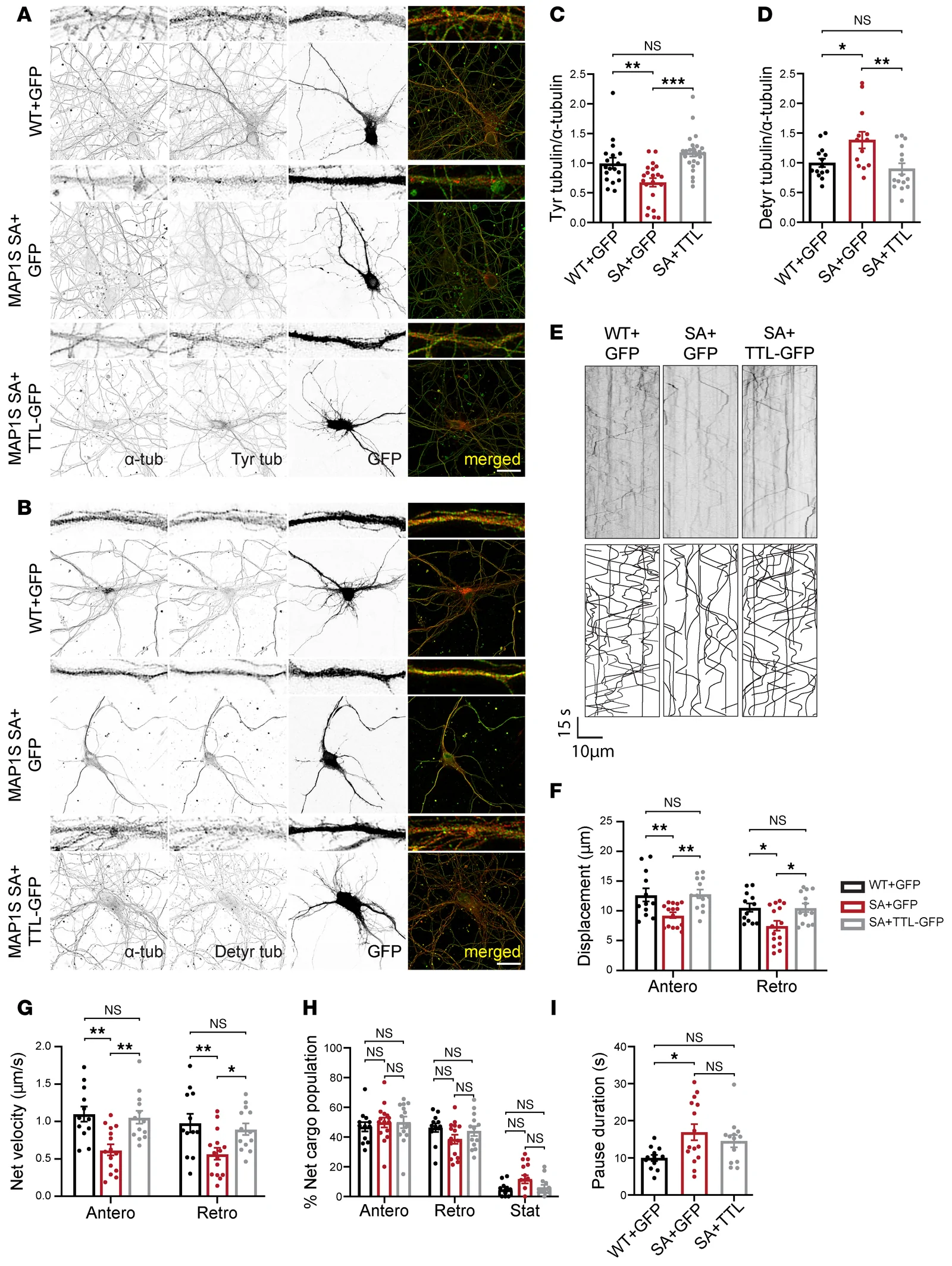
Tubulin tyrosine ligase reexpression rescues dynein motility defects in MAP1S phosphomutant neurons. (**A** and **B**) Immunostainings for tyrosinated and detyrosinated tubulin after GFP or TTL-GFP expression in WT and MAP1S SA neurons. Scale bars: 20 μm. (**C** and **D**) Quantification of **A** and **B**, respectively). WT + GFP, *n =* 14–18 cells, MAP1S SA + GFP, *n =* 13–22 cells, MAP1S SA + TTL-GFP, *n =* 15–26 cells from 2 independent experiments. (**E**–**I**) Live imaging of dynein-pex transport in GFP- or TTL-GFP transfected neurons at DIV7. (**E**) Kymographs. Horizontal scale bar: 10 µm, vertical scale bar: 15 seconds. (**F**) Displacement. (**G**) Net velocity. (**H**) Percentage of net cargo populations. (**I**) Pause duration. WT + GFP, *n =* 12 cells, MAP1S SA + GFP, *n =* 15 cells, MAP1S SA + TTL-GFP, *n =* 13 cells from 2 independent experiments. One-way ANOVA was used to assess statistical significance. *P < 0.05, **P < 0.01, ***P < 0.001. Data are represented as mean ± SEM.

**Figure 6:**
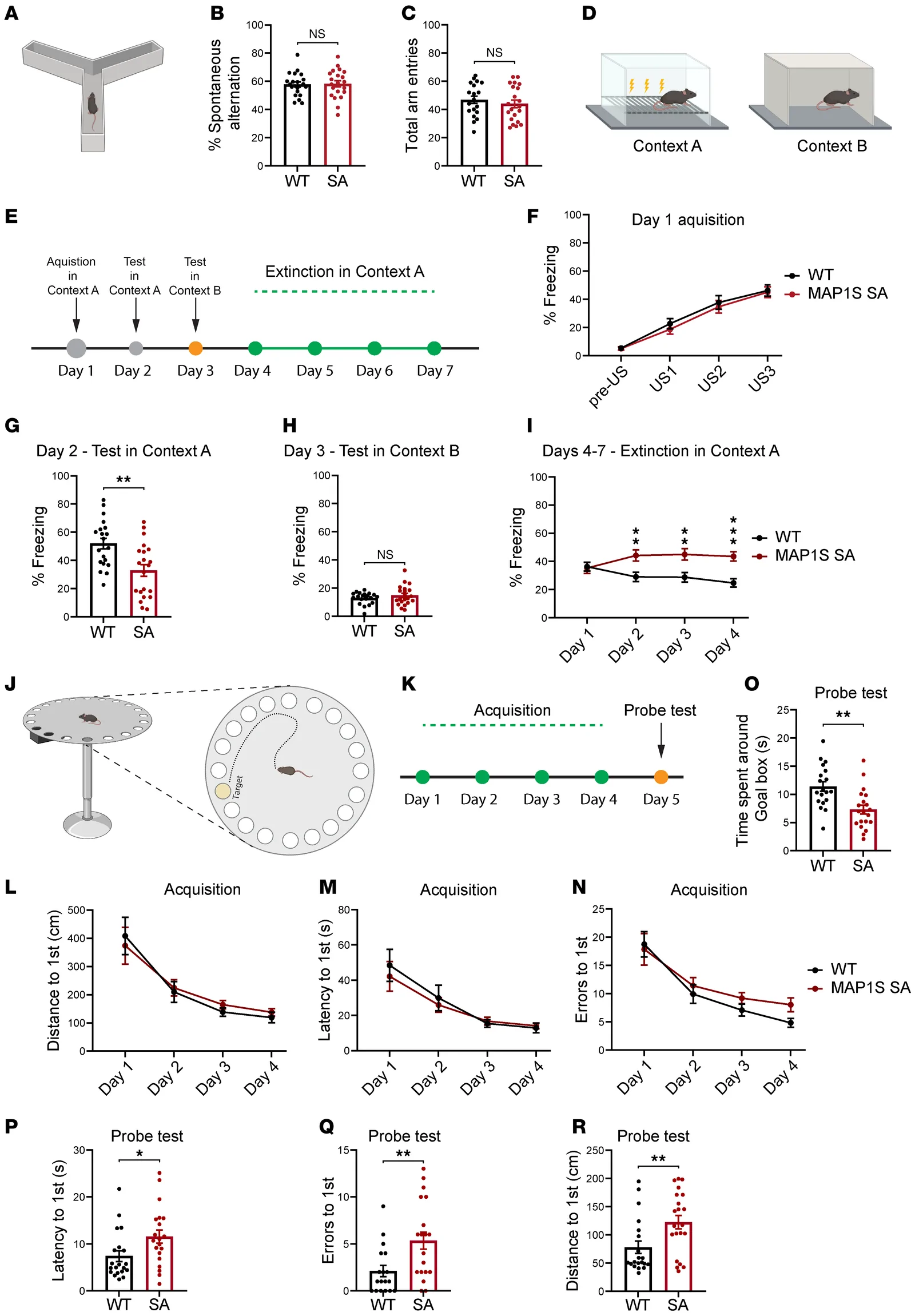
MAP1S phosphorylation regulates associative and spatial reference memory. (**A**–**C**) Spontaneous alternation in the Y-maze using 3-month-old mice. (**B**) Percent alternation between arm entries measures short-term spatial recognition. (**C**) Explorative behavior, revealed by overall number of arm entries in the Y-maze using 3-month-old mice. (**D**) Contextual fear conditioning paradigm. (**E**) Experimental design of the contextual fear conditioning experiment. (**F**) Day 1 acquisition in context A. Percentage of time freezing, indicative of the context/shock association. (**G**) Day 2 test in context A. Percentage of time freezing, indicative of the conditioned fear memories. (**H**) Percentage of time freezing at day 3, context B. On day 3, the animals were placed in a neutral context (context B) without the conditioned stressor (foot shock) and with different sensorial/visual cues. Consequently, the freezing behavior was substantially lower compared with day 2. (**I**) Days 4–7 extinction of the fear memory in context A. From day 4 onward, the mice were reexposed to context A without receiving a foot shock. As a result, WT mice gradually relearned that context A no longer predicted the aversive stimulus, leading to a gradual decline in the defensive freezing behavior. Percentage of time freezing. (**J**) Barnes maze test. (**K**) Experimental design of Barnes maze test. (**L**) Distance traveled to reach goal box. (**M**) Latency to reach goal box during acquisition phase. (**N**) Errors made to reach goal box. (**O**) Time spent around goal box in probe test. (**P**) Latency to first (s). (**Q**) Errors to first. (**R**) Distance to first (cm). WT males, *n =* 10; WT females *n =* 10; MAP1S SA males, *n =* 11; MAP1S SA females, *n =* 10. Two-tailed Student’s *t* test, 2-way ANOVA and 3-way ANOVA were used to assess statistical significance. *P < 0.05, **P < 0.01, ***P < 0.001. Data are represented as mean ± SEM. Cartoons created using BioRender.com.

## Version 1 09/24/2026

In-Press Preview

## Version 2 11/02/2026

Electronic publication
