# Supplementary material for: Microtubule-associated protein 1S phosphorylation regulates dynein-driven transport and synaptogenesis in neurons
Source: J Clin Invest. 2026 Sep 15;136(21):e188241. doi: 10.1172/JCI188241 (PMC13722393; doi:10.1172/JCI188241)

Full scan for Figure 1A

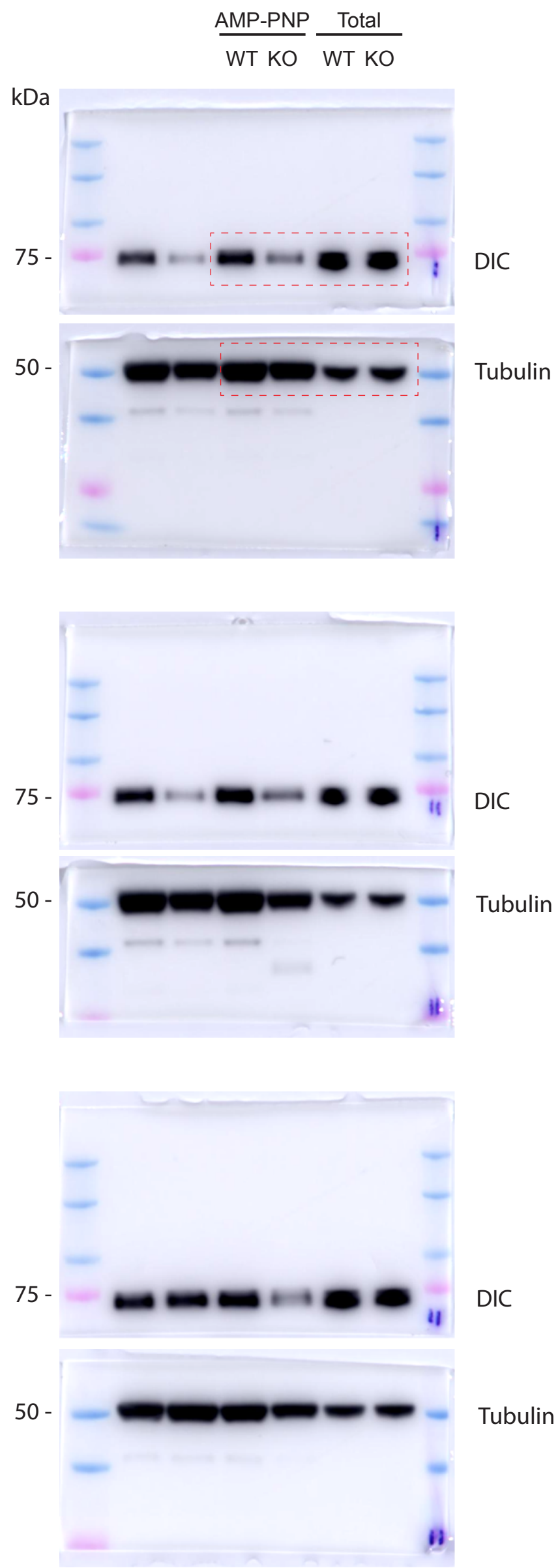

Full scan for Figure 1B

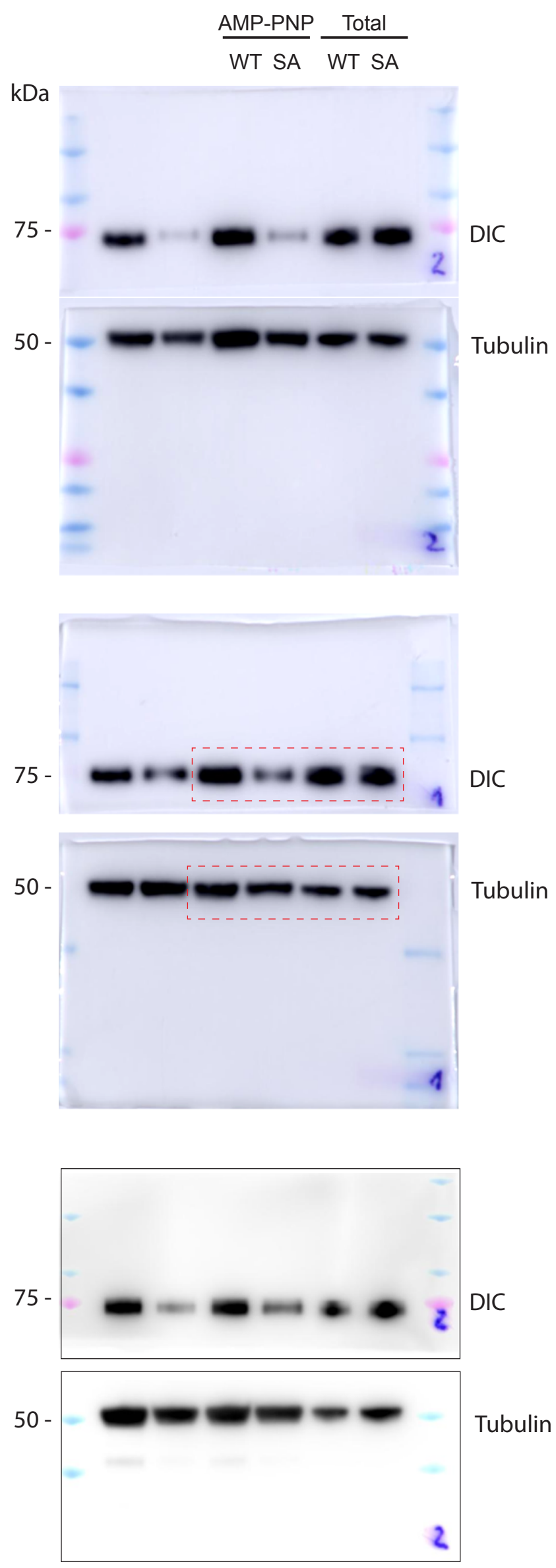

Full scan for Figure 3D - S1 fraction

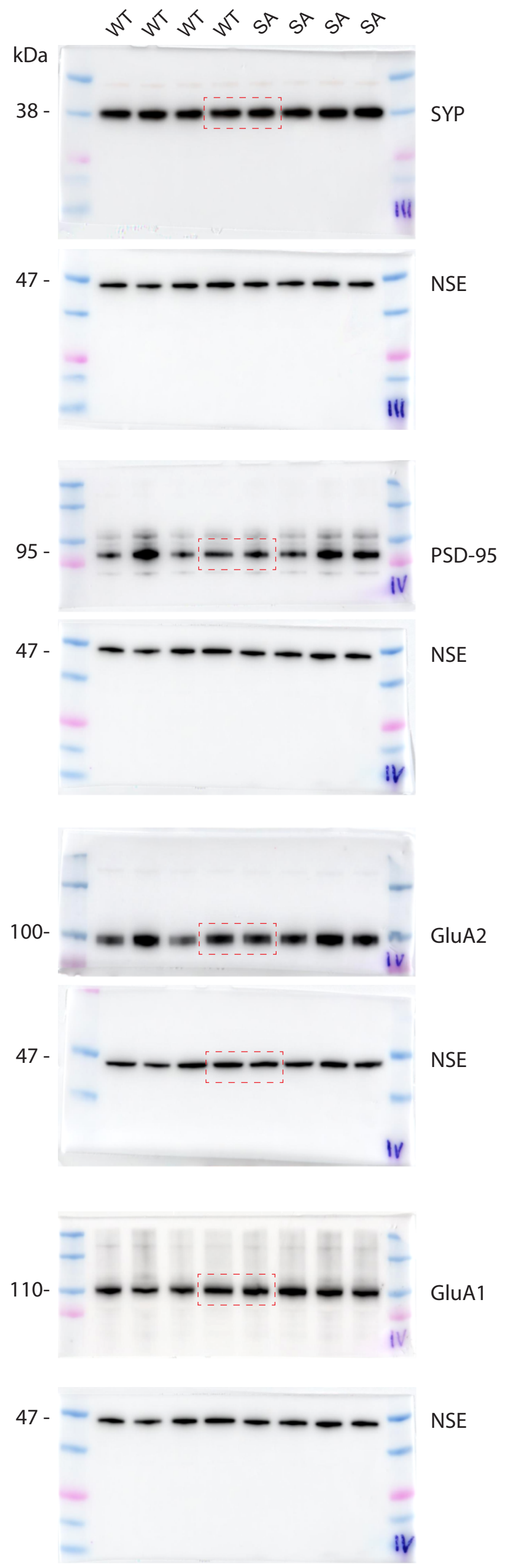

Full scan for Figure 3D - P2 fraction

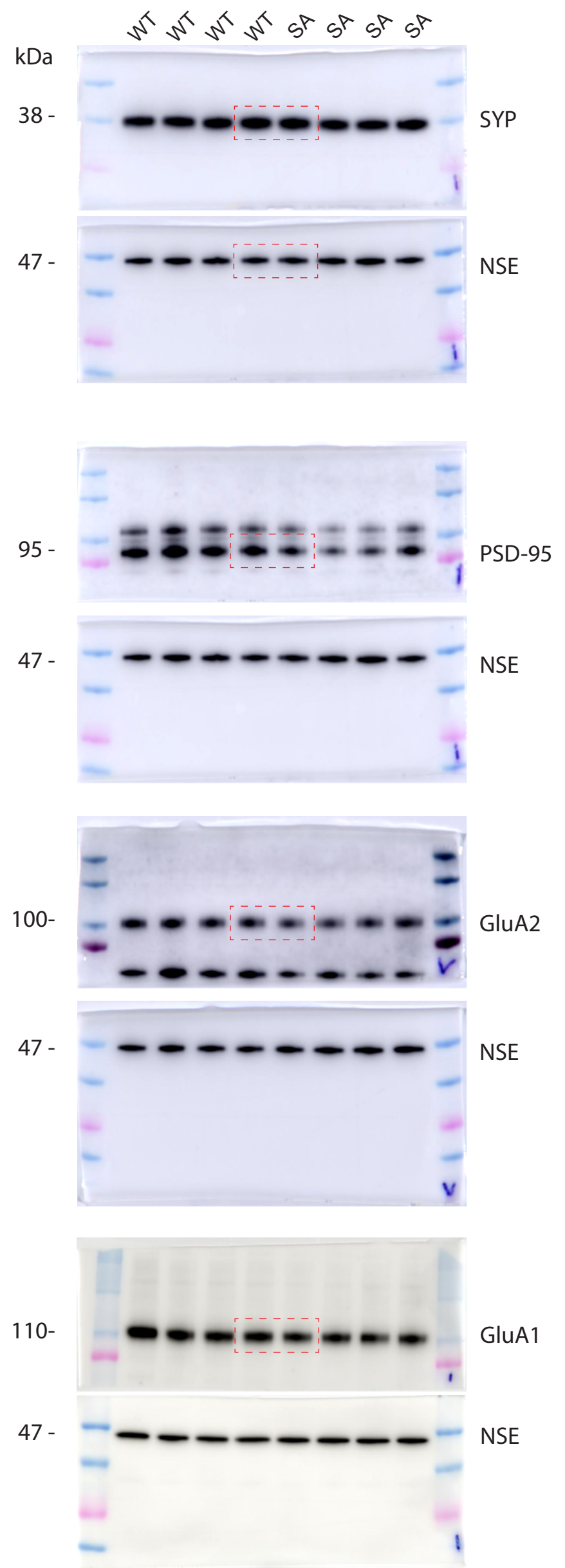

Full scan for Supplemental Figure 1A

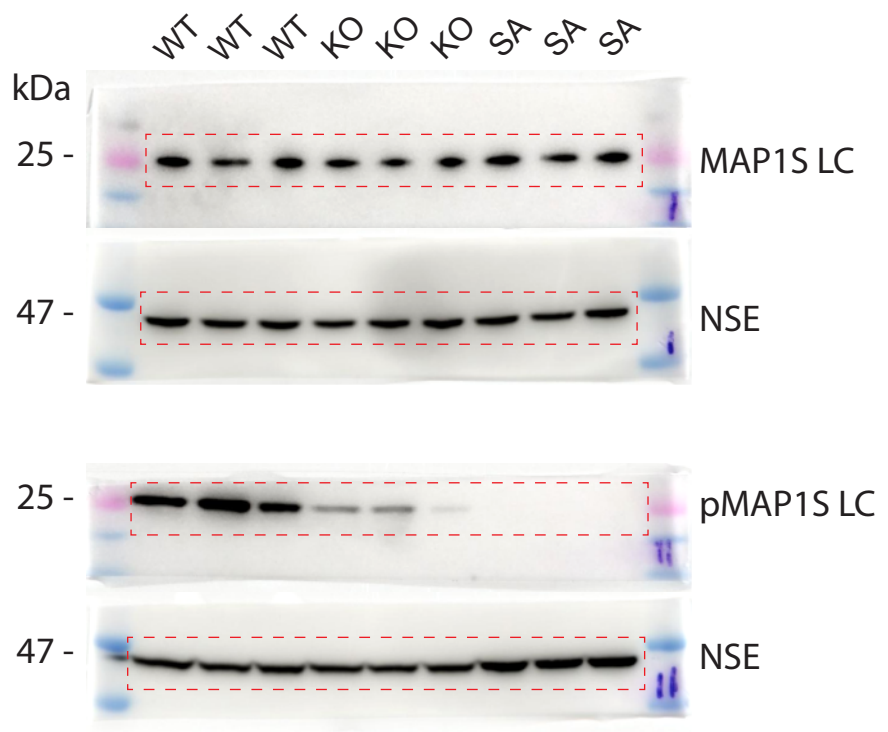

Full scan for Supplemental Figure 1C

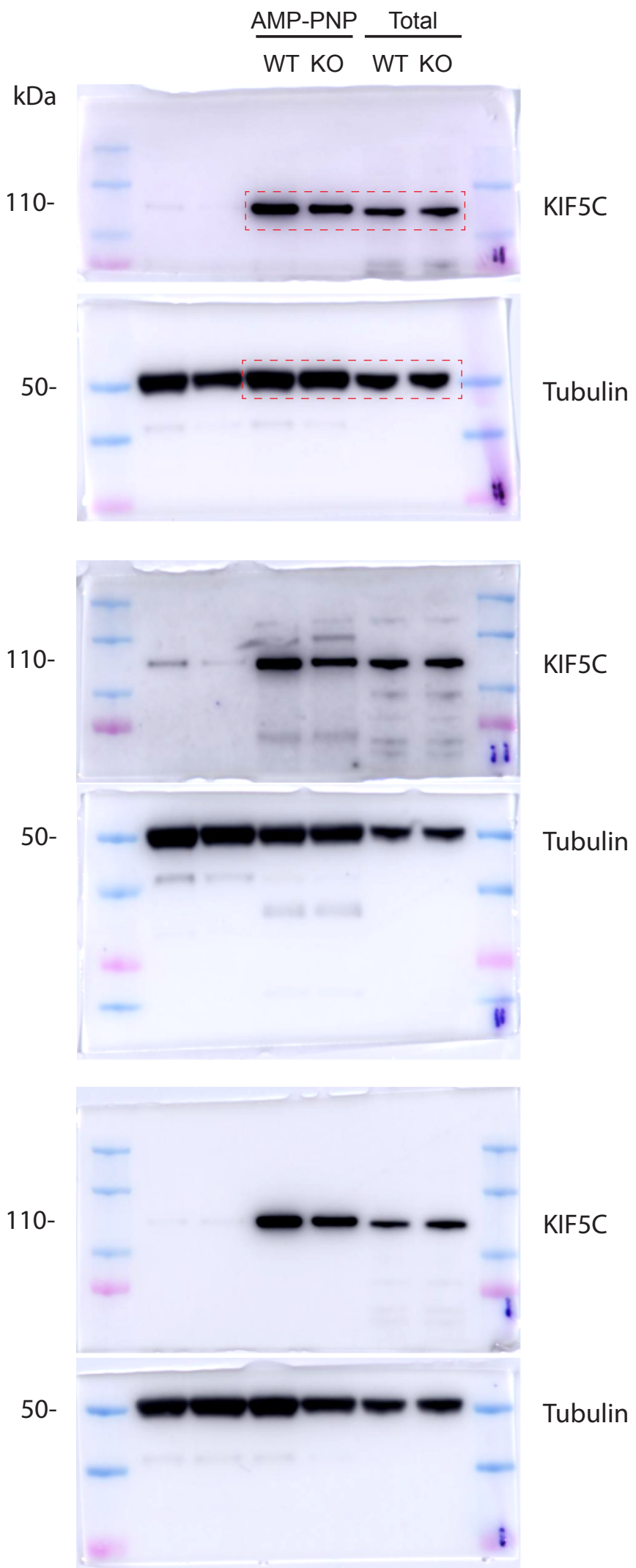

Full scan for Supplemental Figure 1D

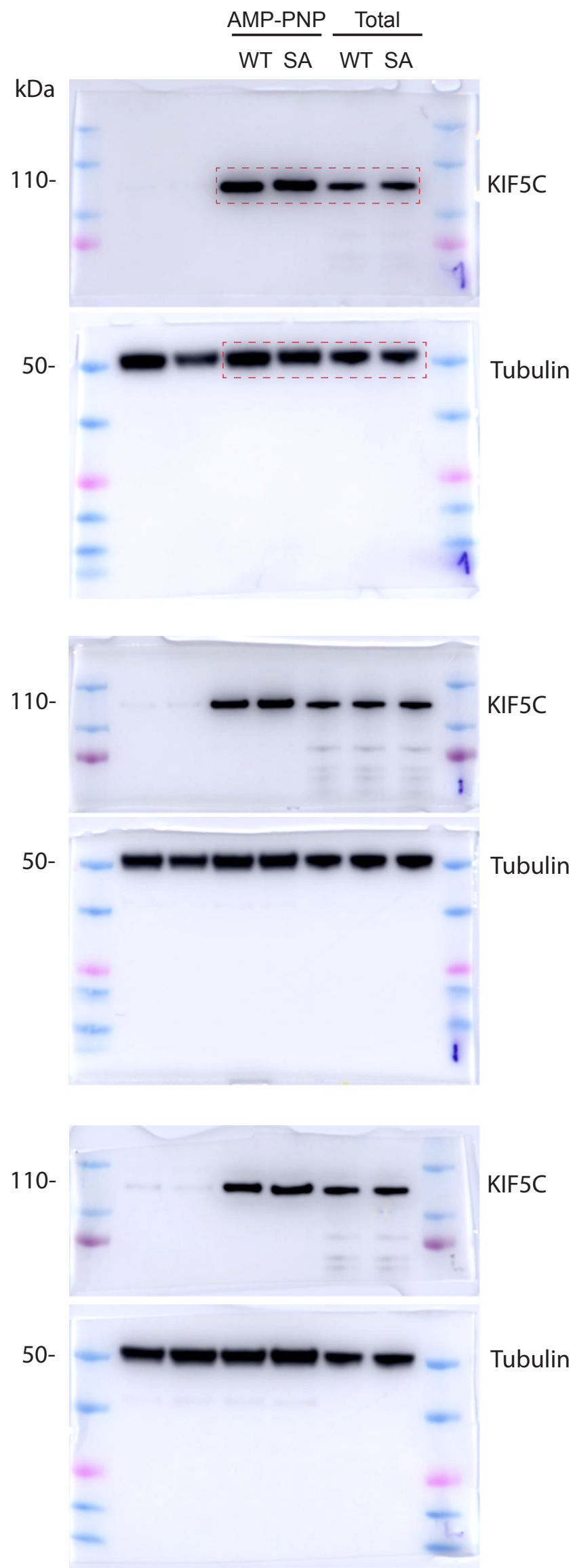

Full scan for Supplemental Figure 1G

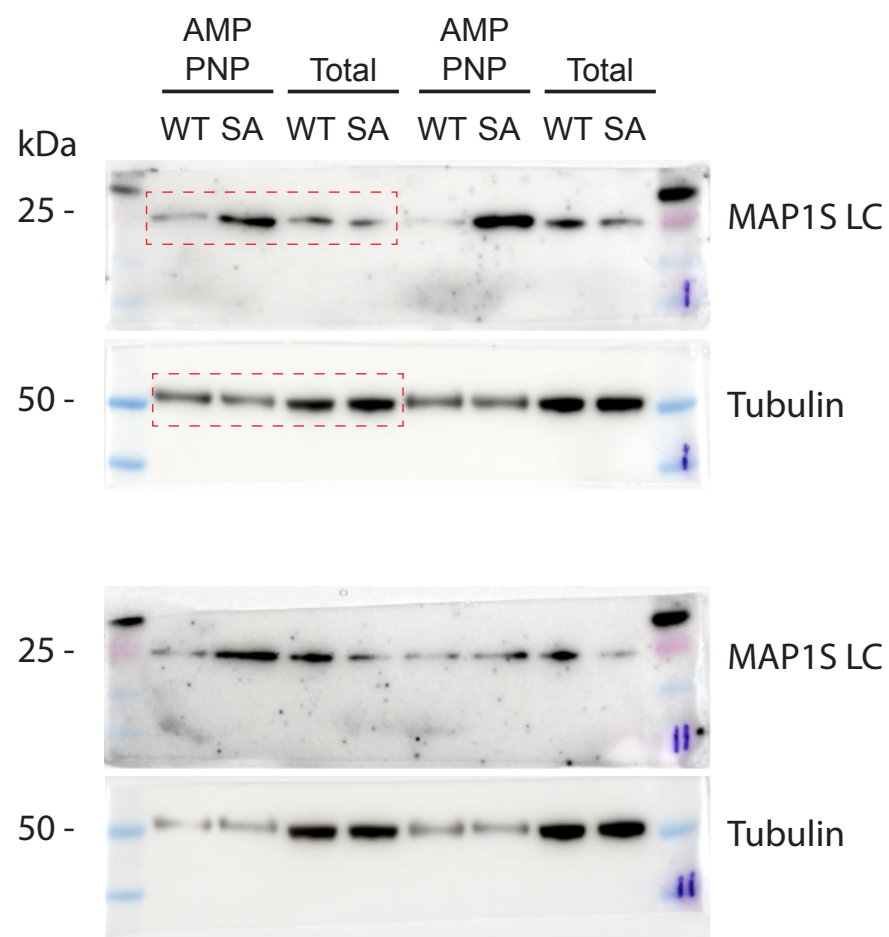

Full scan for Supplemental Figure 2E

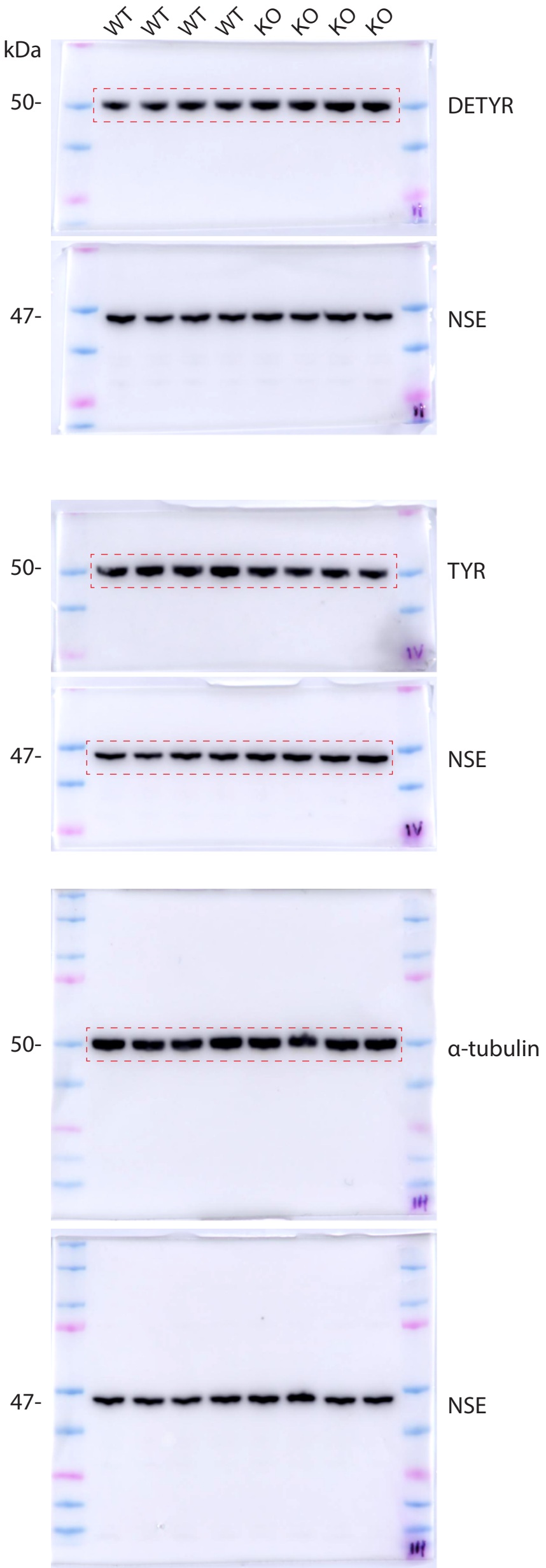

Full scan for Supplemental Figure 2F

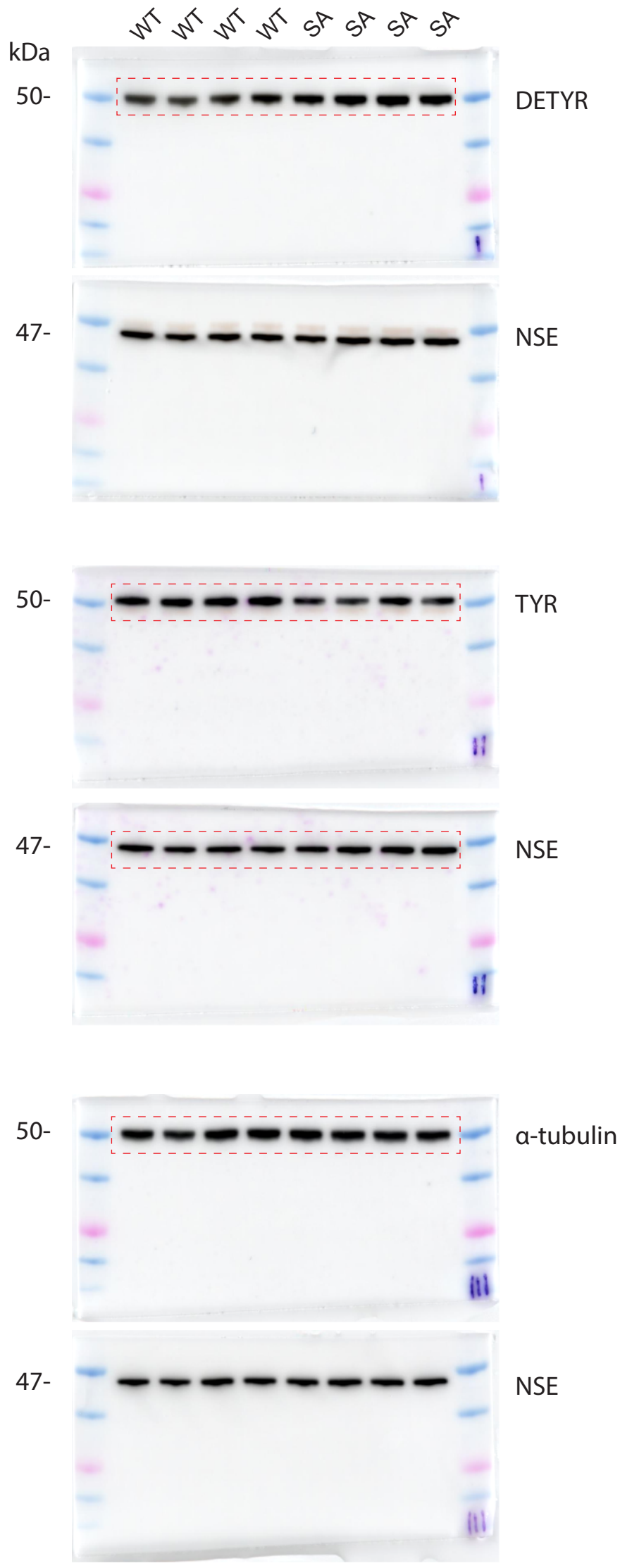

Full scan for Supplemental Figure 3A

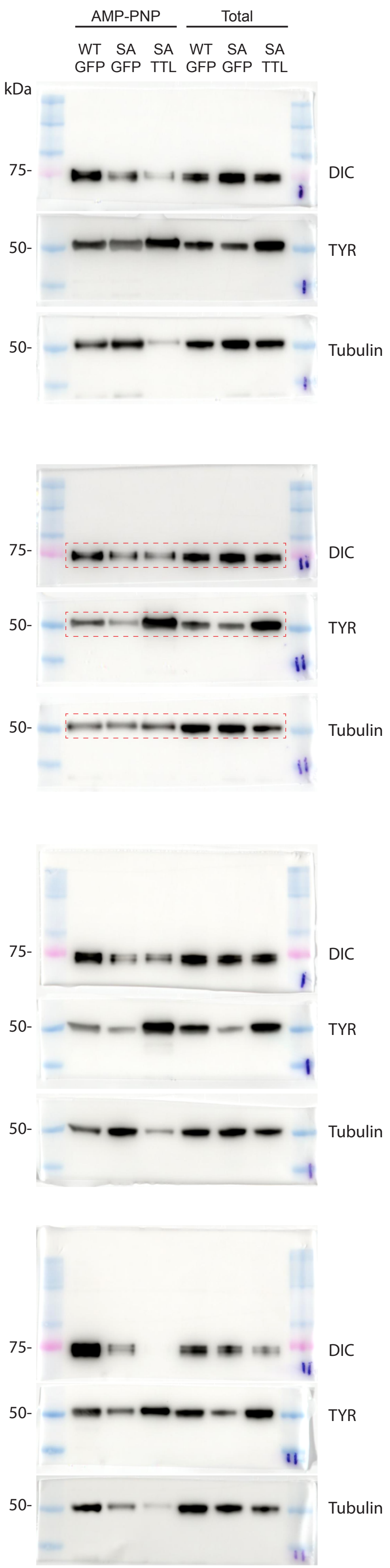

Supplement: Supplementary file 2 [file jci-136-188241-s253.pdf]
